# Comparative transcriptome profiling of two *Brassica napus* cultivars under chromium toxicity and its alleviation by reduced glutathione

**DOI:** 10.1186/s12864-016-3200-6

**Published:** 2016-11-07

**Authors:** Rafaqat A. Gill, Basharat Ali, Peng Cui, Enhui Shen, Muhammad A. Farooq, Faisal Islam, Shafaqat Ali, Bizeng Mao, Weijun Zhou

**Affiliations:** 1 Institute of Crop Science and Zhejiang Key Laboratory of Crop Germplasm, Zhejiang University, Hangzhou, 310058 China; 2 Institute of Biotechnology, Zhejiang University, Hangzhou, 310058 China; 3 Institute of Crop Science and Resource Conservation (INRES), University of Bonn, Bonn, 53115 Germany; 4 Department of Environmental Sciences, Government College University, Faisalabad, 38000 Pakistan

**Keywords:** *Brassica napus* L, Chromium, Reduced glutathione, Transcriptome profile analysis, Digital gene expression, Transcription factors, Proteome analysis

## Abstract

**Background:**

Chromium (Cr) being multifarious industrial used element, is considered a potential environmental threat. Cr found to be a prospective water and soil pollutant, and thus it is a current area of concern. Oilseed rape (*Brassica napus* L.) is well known as a major source of edible oil around the globe. Due to its higher growth, larger biomass and capability to uptake toxic materials *B. napus* is considered a potential candidate plant against unfavorable conditions. To date, no study has been done that described the Cr and GSH mechanism at RNA-Seq level.

**Results:**

Both digital gene expression (DGE) and transcriptome profile analysis (TPA) approaches had opened new insights to uncover the several number of genes related to Cr stress and GSH alleviating mechanism in two leading cultivars (ZS 758 and Zheda 622) of *B. napus* plants. Data showed that Cr inhibited KEGG pathways i.e. stilbenoid, diarlyheptanoid and gingerol biosynthesis; limonene and pentose degradation and glutathione metabolism in ZS 758; and ribosome and glucosinolate biosynthesis in Zheda-622. On the other hand, vitamin B6, tryptophan, sulfur, nitrogen and fructose and manose metabolisms were induced in ZS 758, and zeatin biosynthesis, linoleic acid metabolism, arginine and proline metabolism, and alanine, asparate and glutamate metabolism pathways in Zheda 622. Cr increased the TFs that were related to hydralase activity, antioxidant activity, catalytic activity phosphatase and pyrophosphatase activity in ZS 758, and vitamin binding and oxidoreductase activity in Zheda 622. Cr also up-regulated the promising proteins related to intracellular membrane bounded organelles, nitrile hyrdatase activity, cytoskeleton protein binding and stress response. It also uncovered, a novel Cr-responsive protein (CL2535.Contig1_All) that was statistically increased as compared to control and GSH treated plants. Exogenously applied GSH successfully not only recovered the changes in metabolic pathways but also induced cysteine and methionine metabolism in ZS 758 and ubiquinone and other terpenoid-quinone biosynthesis pathways in Zheda 622. Furthermore, GSH increased the level of TFs i.e. the gene expression of antioxidant and catalytic activities, iron ion binding and hydrolase activity as compared with Cr. Moreover, results pointed out a novel GSH responsive protein (CL827.Contig3_All) whose expression was found to be significantly increased when compared than Cr stress. Results further delineated that GSH induced TFs such as glutathione disulphide oxidoreducatse and aminoacyl-tRNA ligase activity, and beta glucosidase activity in ZS 758. Similarly in Zheda 622, GSH induced the TFs for instance DNA binding and protein dimerization activity. GSH also highlighted the proteins that were involved in transportation, photosynthesis process, RNA polymerase activity, and against the metal toxicity. These results indicated that cultivar ZS 758 had better metabolism and showed higher tolerance against Cr toxicity.

**Conclusion:**

The responses of ZS 758 and Zheda 622 differed considerably at both physiological and transcriptional level. Moreover, RNA-Seq method explored the hazardous behavior of Cr as well as GSH up-regulating mechanism by activating plant metabolism, stress responsive genes, TFs and protein encyclopedia.

**Electronic supplementary material:**

The online version of this article (doi:10.1186/s12864-016-3200-6) contains supplementary material, which is available to authorized users.

## Background

Chromium (Cr) is a metallic element that found in large quantity on earth crust. Cr being 22nd ranked element on the world’s soil, is available in various concentrations ranges from 1 to 300 mg/kg with an average of 100 mg/kg [1, 2]. Thus, it is available in different reactive forms, but promising species are monovalent (Cr 0), trivalent (Cr III), and the hexavalent (Cr VI) [3]. It comes into the plant body passively [4] and also through the sulphate anions as a carrier [5]. It is unspoken that Cr may not involve in any physiological processes in plants but it negatively regulates its functional behavior [6, 7]. Toxicity of Cr and other metals influences the multiplicity of processes in plants by induction of cell structural damages in both leaf and root cells, and increases the metal uptake in various plant organs [7–10]. Prominent changes induced by heavy metal are the generation of reactive oxygen species (ROS), that are involved in deterioration of plant physio-molecular attributes by the direct damages of nucleic acid, chloroplast structures and cell membranes [11, 12]. Enhanced level of ROS in different plant parts might be the imbalance between its production and inactivation by antioxidants that known as superoxide dismutase, peroxidase, catalase and ascorbate peroxidase, and also non enzymatic defense machinery called glutathione and carotenoids [13, 14].

Along with these antioxidants, exogenous application of growth promoters is recently been a field of investigation. One of the promising ones, reduced glutathione (GSH) is known as a ROS alleviator under the heavy metal stress in plants [15]. Being a non-protein based antioxidant, it has defined role to scavenge the photosynthetic by-products, the ROS in cell organelles and also wipeout the activated oxygen species with the help of ascorbate [16]. Furthermore, GSH plays a key role as a substrate for phytochelatins, which are implicated to detoxify the metal toxicity in plants [17, 18]. Besides, GSH is highly soluble in water; this property makes it promising growth regulator against the metal toxicity, including ROS scavenging and xenobiotics [19, 20].

With the advancement in molecular approaches such as DGE and TPA analyses, to better insights the biological phenomena of living thing have become achieved. For instance, comparative RNA-seq analyses between different cultivars and among treatments can make available the deep understanding into the actual metabolic pathways. Recently, sorghum transcriptomic encyclopedia explored the large amount of stress responsive genes [21, 22]. Moreover, Zeng et al. [23] explored the barley genome under the low potassium stress by using RNA-Seq method. Development of these metabolic databases largely facilitated the studies focusing on metal tolerant. Thus, next generation sequence could be a promising technique to figure out the genomics and biochemical behavior involved in *Brassica napus* under the Cr stress alleviated by exogenously applied GSH.

As for oilseed rape (*Brassica napus* L.) plants, recent studies have concentrated its capability to respond the heavy metal stress, uptake and distribution of metal contents, effect on photosynthetic gas exchange capacity, biochemical and ultrastructural changes [7, 24, 25]. According to our knowledge there is no report which reveals the DGE and TPA analyses under the Cr stress mitigated by GSH. Hence, the present study was carried out to explore the *B. napus* behavior by using the RNA sequence analyses against Cr stress and its alleviation through exogenous GSH.

## Methods

### Plant material and growth conditions

For this study, seeds of two leading cultivars of *B. napus* named as black seeded (ZS 758) and yellow seeded (Zheda 622) were selected on the basis of previous findings [10], in which we found that these two cultivars showed different tolerance response to Cr stress. Seeds of these two cultivars were collected from Zhejiang University, Hangzhou, China. Mature seeds were washed with distilled H_2_O thoroughly. In every Petri dish, 60 seeds were positioned for overnight at a wet filter paper. Total, 30 seedlings were chosen randomly for each treatment and transferred to plastic box (12 cm^2^, having sponge inside) after germination. Uniform seedlings were subjected to different combinations of Cr and GSH (0 μM, 400 μM Cr and 400 μM Cr + 1.0 mM GSH) and allowed them for six days. These treatments repeated twice, second treatment was applied three days later after the first treatment. Here, Cr concentration was selected on the basis of our previous work [10]. The GSH concentration was selected after the preliminary experiment in which we used 0, 0.5, 1, 1.5 and 2.0 mM concentrations and we found that 1.0 mM GSH performed better and was added in Cr solution. The potassium dichromate (K_2_Cr_2_O_7_) salt was utilized to establish various Cr levels in solution, and full-strength Hoagland’s solution was used as a basal medium with three replications. Experiment was performed in a controlled chamber with the day/night temperatures of 24/16 °C, a 16-hr photoperiod, irradiance of 300 μmol m^−2^ s^−1^, and relative humidity of 60–70 %. Plants were harvested and separated into shoot and root for the measurement of dry weight after six days. The leaf samples were used for RNA- Seq analyses.

### Estimation of dry matter, Cr and GSH contents

Dry matter contents of shoots and roots per plant were measured according to Zhang et al. [26]. Cr contents were estimated according to Gill et al. [25] and for GSH contents the methodology of Law et al. [27] was followed with some modifications [25].

### Total RNA extraction, reliability assessment and RNA-sequence analyses

Total RNA was extracted manually from six samples of both cultivars with TRIzol regent (Invitrogen, USA) according to the manufacturer’s instruction. Integrity of samples was verified by minimum RNA integrated number of 8 by the 2100 bio-analyzer (Agilent). Then RNA samples were first treated with DNase I (Takara Biotechnology, China) to degrade any possible DNA contamination. Then the mRNA was enriched by using the oligo (dT) magnetic beads (for eukaryotes). After that fragmentation buffer was added to fragment the mRNA into short pieces (about 200 bp). Then the first strand of cDNA was synthesized by using random hexamer-primer. Buffer, dNTPs, RNase H and DNA polymerase I added to synthesize the second strand. The double strand cDNA was purified with magnetic beads. End reparation and 3’-end single nucleotide A (adenine) addition was then performed. Finally, sequencing adaptors were ligated to the fragments. The fragments are enriched by PCR amplification. During the QC step, Agilent 2100 Bio-anaylzer and ABI StepOnePlus Real-Time PCR System were used to qualify and quantify the sample library. The library products are ready for sequencing via Illumina HiSeq^TM^ 2000 or other sequencer when necessary.

### Establishment of *de novo* assembly

Both DGE and TPA analyses generated total raw reads were cleaned from adapter sequences, mismatch and low quality reads with the help of internal software called filter_fq and then data saved as “.fastq” files. To avoid the error rate from the sequences we used the following formula, where E denoted the error rate and SQ to the base quality of Illunima HiSeqTM 2000.

1 $$ \begin{array}{c}\hfill \begin{array}{c}\mathrm{S}\mathrm{Q}=-10\times \left( \log \frac{\mathrm{E}}{1-\mathrm{E}}\right)/\left( \log 10\right)\\ {}\mathrm{E}=\frac{\mathrm{Y}}{1+\mathrm{Y}}\end{array}\hfill \\ {}\hfill \mathrm{Y}=\frac{\mathrm{SQ}}{{}_e-10\times log10}\hfill \end{array} $$

After that, clean reads were mapped to reference sequences set using SOAPaligner/SOAP2 method [28], http://soap.genomi cs.org.cn/soapaligner.html. Expression level for each gene was calculated by using reads per kb per million reads (RPKM) method [29]; the suggested formula is shown as follows:

2 $$ RPKM=\frac{10^6C}{NL/{10}^3} $$

Here RPKM is the expression level of gene, C is number of reads that uniquely aligned to gene, N is total number of reads that uniquely aligned to all genes, and L is number of bases of gene.

### Screening of differentially expressed genes (DEGs), group differentially expressed genes, and RT-PCR analysis

DEGs that produced by DGE and TPA were screened according to Audic and Calverie [30] with some modifications. This method recommended the strict algorithm to identify the DEGs between two samples, equation is given below:

3 $$ p\left(\times \right)=\frac{e^{-\lambda }{\lambda}^x}{x!}\left(\lambda\ \mathrm{is}\ \mathrm{the}\ \mathrm{real}\ \mathrm{transcripts}\ \mathrm{of}\ \mathrm{the}\ \mathrm{gene}\right) $$

Where x represent the gene that occupy only a small portion of library and p(x) closely follow the Poisson distribution. We calculated the probability of equally expressed genes by using the following formulas;

4 $$ \begin{array}{l}2{\displaystyle \sum_{i-0}^{i-y}p\left(i\left|x\right.\right)}\\ {}\mathrm{or}\kern0.5em 2\times \left(1-{\displaystyle \sum_{i-0}^{i-y}p\left(i\left|x\right.\right)}\right)\kern1em \left(\mathrm{if}{\displaystyle \sum_{i-0}^{i-y}p\left(i\left|x\right.\right)>0.5}\right)\\ {}p\left(y\left|x\right.\right)=\left(\frac{N_2}{N_1}\right)y\frac{\left(x+y\right)!}{x!y!\left(1+\frac{N_2}{N_1}\right)\left(x+y+1\right)}\end{array} $$

Where, N1 is the clean tag number of sample 1 and N_2_ to sample 2; gene A holds x tags in sample1 and y tags in sample 2, and *P*-value corresponds to differential gene expression test. To determine the threshold of *P*-value in multiple samples, the FDR (False Discovery Rate) method was employed [31]. Group differentially expressed genes were screened by the NOIseq method [32]. According to this method, we made noise distribution of the actual data. First, we used sample’s gene expression in each group to calculate log_2_ (fold change) M and absolute different value D of all pair conditions (gene expression value will be substituted by 0.001 if it doesn’t express in some sample). Second, average expression value of each gene standing for replicates was used to calculate M and D. Two replicates in one of the experimental conditions are sufficient to run the algorithm.

5 $$ {M}^i = \mathrm{l}\mathrm{o}{\mathrm{g}}_2\left(\frac{x_1^i}{x_2^i}\right)\ \mathrm{and}\ {D}^i=\left|{x}_1^i-{x}_2^i\right| $$

**Fig. 1:**
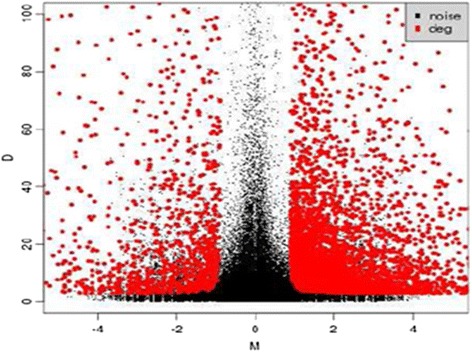
M-D noise distribution model

Then, all these M/D values are pooled together to generate the noise distribution that is shown in Fig 1. In order to verify the Hiseq 2000™ data both RNA and RNA-Seq samples were used, and we followed the previous methodology of Gill et al. [25].

**Fig. 2:**
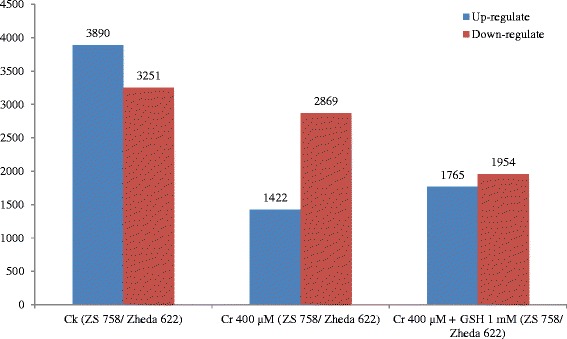
Comparative number of differentially expressed genes analyzed by digital gene expression analysis. Three following treatments were used i.e. control (Ck), Cr 400 μM, and Cr 400 μM + GSH 1 mM. Cultivar ZS 758 considered as control, while cultivar Zheda 622 as treatment

### Expression pattern analysis of DEGs

We performed cluster analysis to display gene expression patterns with the help of Cluster [33] software and Java TreeView [34] software. In this analysis, each row represented a gene and expression differences are shown in different colors. Red means up regulation and green means down regulation.

### Gene Ontology (GO) functional enrichment analysis (WEGO) of DEGs

In order to calculate the go domains we used the Blast2GO [35] software, then all DEGs blast to the GO database (http://www.geneontology.org/). We calculated the gene numbers for every term by using hyper-geometric test and explore the significantly enriched GO terms in DEGs comparing to the genome background. The calculating formula is:

6 $$ \mathrm{P}=1-{\displaystyle \sum_{i=0}^{m-1}\frac{\left(\underset{i}{M}\right)\left(\underset{n-i}{N-M}\right)}{\left(\underset{n}{N}\right)}} $$

Where N is the number of all genes with GO annotation; n is the number of DEGs in N; M is the number of all genes that are annotated to the certain GO terms; m is the number of DEGs in M. The calculated *p*-value referred to Bonferroni correction, we took corrected *p-value ≥0.05* as a threshold. We only selected those GO terms which fulfilling this condition and are defined as significantly enriched GO terms in DEGs. After getting GO annotation for DEGs, we used WEGO [36] software in order to elaborate the GO functional classification for DEGs and to understand the distribution of gene functions of *B. napus* on macro level.

### KEGG pathway enrichment analysis

Similar to GO analysis we used the same software (Blast2GO) and same formula prior to blast the DEGs with Kyoto Encyclopedia of Genes and Genomes (KEGG) data base, http://www.genome.jp/ [37]. In that formula, for KEGG analysis, N is the number of all genes that with KEGG annotation, n is the number of DEGs in N, M is the number of all genes annotated to specific pathways and m is number of DEGs in M.

### Identification of protein-protein interactions networks

In order to estimate the protein-protein interaction networks among DEGs that coding proteins in both cultivars and treatments we used “Cytoscape” software according to earlier described method [38], http://www.ncbi.nlm.nih.gov/pubmed/23132118. Prior to “Cytoscape” plugin, the selection criteria of the genes coding protein were the correlation coefficient R and FDR values based on the Pearson method. Those DEGs were selected that had the correlation value larger than 0.99 or lesser than −0.99, and the FDR less than 0.05.

### Verification of RNA-Seq data by quantitative real-time PCR (qRT-PCR) assay

Using Trizol method, complete RNA was haul-out from ~100 mg freeze leaf tissues of both cultivars. Prime Script ^TM^RT reagent kit with gDNA eraser was used to erase the genomic DNA and cDNA synthesis. cDNA samples from different treatments were assayed by qRT-PCR in the iCycler iQTM Real-time detection system (Bio-Rad, Hercules, CA, USA) using SYBR® Premix Ex Taq II (Takara Co. Ltd). The software given with the PCR system was used to calculate the threshold cycle values and quantification of mRNA levels was achieved according to the method of Livak and Schmittgen [39]. The primers used for randomly selected stress responsive genes are presented in Additional file 1: Table S3.

### Statistical analyses

The consequence of difference between the two *B. napus* cultivars, both in physiological and RNA-seq attributes was analyzed by using data processing system (DPS), followed by the Duncan’s multiple range test (DMRT). The differences were considered in the form of significance and highly at 0.05 and 0.01 (probability level), respectively.

## Results

### Plant biomass, Cr and GSH contents determination

In current study, data regarding the biomass showed that Cr stress alone significantly reduced the dry matter of leaves and roots in both *B. napus* cultivars (Table 1). Exogenous enrichment of GSH in to the nutrient medium was statistically recovered the negative impacts of metal in both plant organs. Similarly, Cr stress increased the Cr contents in leaves and roots in both cultivars. Besides, it was also analyzed that growth enhancer GSH significantly reduced the Cr contents in both organs; interestingly it further increased the thione activity in roots but slightly decreased in leaf as compared to metal alone. Furthermore, results indicated that ZS 758 was performed better against the above mentioned parameters and showed higher tolerance against Cr toxicity.

**Table 1:** Analyses of dry biomass, chromium (Cr) and reduced glutathione (GSH) contents under different concentrations of Cr (400 μM) and GSH (1 mM) in two cultivars of *Brassica napus*

Cultivar	Treatment	Dry weight (g/plant)		Cr content (mg Kg^−1^ DW)		GSH content (μmol min^−1^ mg^−1^ protein)	
		Leaf	Root	Leaf	Root	Leaf	Root
ZS 758	Ck	0.074 ± 0.002 a	1.8 ± 0.05 a	0.015 ± 0.002 e	0.022 ± 0.002 e	7.13 ± 1.03 c	5.62 ± 0.52 b
	Cr	0.053 ± 0.003 c	1.3 ± 0.15 c	46.31 ± 5.01 c	958.22 ± 53.02 c	11.34 ± 1.04 a	6.48 ± 0.58 ab
	Cr + GSH	0.067 ± 0.002 b	1.66 ± 0.06 a	28.43 ± 2.03 d	614.56 ± 33.76 d	9.62 ± 1.02 ab	7.18 ± 1.08 a
Zheda 622	Ck	0.076 ± 0.002 a	1.75 ± 0.05 a	0.017 ± 0.002 e	0.028 ± 0.002 e	7.06 ± 1.06 c	5.53 ± 0.53 b
	Cr	0.043 ± 0.001 d	0.9 ± 0.05 d	71.94 ± 5.04 a	1372.96 ± 70.06 a	8.14 ± 1.04 bc	5.74 ± 0.54 b
	Cr + GSH	0.051 ± 0.001 c	1.44 ± 0.04 b	52.84 ± 3.04 b	1046.97 ± 56.06 b	7.54 ± 1.04 c	6.68 ± 0.58 ab

Values are the means of three replications ± SD. Variants possessing the same letter are not statistically significant at *P* < 0.05

### RNA sequencing and *de novo* assembly

In this experiment, cDNA from three treatments (Ck, Cr and Cr + GSH) of both cultivars were subjected to Illumina sequencing platform. Total reads by DGE were 11874992, 10905525 and 11759780 in ZS 758 and 11644499, 12509943 and 12148654 in Zheda 622 under Ck, Cr and Cr + GSH, respectively (Additional file 1: Table S1). Total mapped reads were 78 %, 73 % and 78 % in ZS 758 and 79 %, 74 % and 80 % in Zheda 622 under Ck, Cr and Cr + GSH environments, respectively. Under the Cr condition, DGE mapped reads percentages were significantly lower than Ck and Cr + GSH treatments. Moreover, a similar trend was also observed in the cases of perfect match, unique match and multi-position match (Additional file 1: Table S1).

**Fig. 3:**
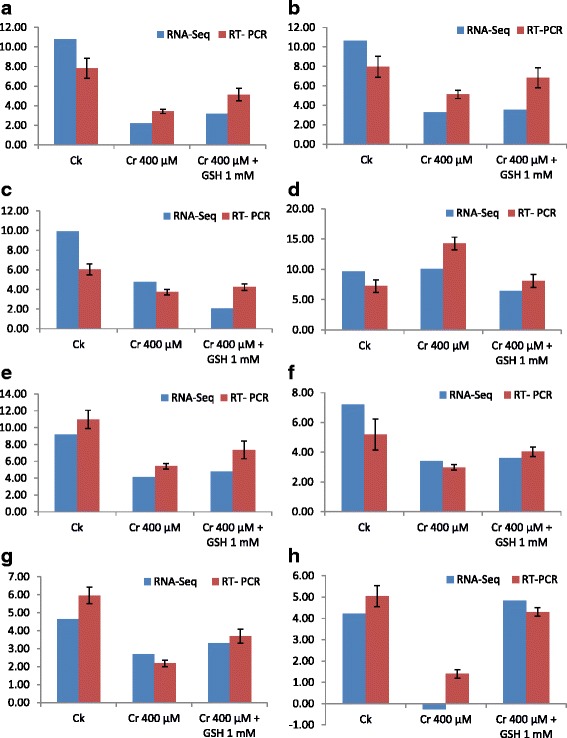
**a**–**h** Comparative analysis of randomly selected genes transcribed by RNA sequence and after that verified by real time PCR analyses. These genes description are follows i.e. oxidoreductase activity (**a**), Antioxidant activity and transition metal ion binding (**b**), chlorophyll A-B binding and early light-inducible protein (**c**), predicted protein (**d**), hypothetical protein (**e**), hypothetical protein (**f**), cofactor binding and transferring hexosyl groups (**g**) and hydrolase activity and hydrolyzing O-glycosyl compounds (**h**), respectively under the Ck, Cr 400 μM, and Cr 400 μM + GSH 1 mM conditions

**Fig. 4:**
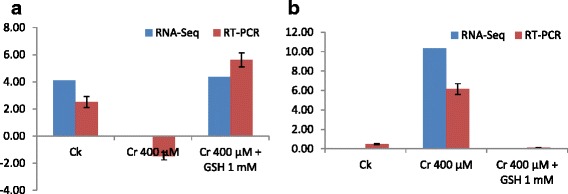
**a**, **b** Shows the randomly selected transcripts that were generated by RNA sequence and verified by RT-PCR analyses. These transcripts are peroxidase 52 (**a**), and Unknown protein (**b**), respectively under the Ck, Cr 400 μM, and Cr 400 μM + GSH 1 mM treatments conditions

Similarly, data regarding the TPA were also performed and, results were obtained in the form of contigs (CLs) and unigenes (Us) (Additional file 1: Table S2). There was 245,841 CL in ZS 758 with a mean length of 267 bps and 220,271 CLs in Zheda 622 with the average of 280 bps. Besides, more than 3000 nts (nucleotides) size was 578 bps in ZS 758 and 620 bps to Zheda 622. Moreover, we found 109,189 total unigenes (ZS 758 + Zheda 622) from both cultivars that were 91620 in ZS 758 (with 762 bps mean value) and 86328 to Zheda 622 (with 731 bps mean value). Besides, it was also observed that data regarding the unigenes size >3000 nts were 928 bps in ZS 728, 1124 bps in Zheda 622 and 1647 bps to the total Us. Results showed that number of Us were more in ZS 758 as compared to Zheda 622. Furthermore, at 200–300 nts Us difference was quite obvious between two cultivars i.e. 28433 bps in ZS 758 and 25562 bps in Zheda 622. Interestingly, this difference became equal at 1000 nts but again at >3000 nts were more obvious in Zheda 622 (1124 bps) as compare to ZS 758 (928 bps). Furthermore, clean reads were mapped by using *de novo* sequencing (Additional file 2: Figure S1) and then subjected to BLAST2GO (BLAST2GO2.2.5, software) against the non-redundant (nr) nucleotide database (website, http://blast.ncbi.nlm.nih.gov/Blast.cgi) in order to obtain the gene ontology (GO). To verify the Illumina platform (Hiseq™ 2000) results, we selected the ten random differentially expressed genes (DEGs) (that were common in both cultivars) and designed the paired primers of relevant genes for RT-PCR. In this study, all paired primers of selected DEGs (Additional file 1: Table S3) validated the RNA- Seq results (Figs. 3a–h, 4a, b). These randomly selected transcripts were related to stress response i.e. oxidoreductase activity, antioxidant activity and transition metal ion binding, chlorophyll A-B binding and early light-inducible protein, cofactor binding and transferring hexosyl groups, hydrolase activity and hydrolyzing O-glycosyl compounds and peroxidase 52.

### Screening, expression pattern analysis and clustering of differentially expressed genes (DEGs)

Whole *B. napus* (ZS 758 and Zheda 622) leaf genome was uncovered via DGE and TPA techniques. Additional file 2: Figure S2 shows the number of up and down regulated genes in the form of scattered plots under Ck, Cr and Cr + GSH. Up-regulated DEGS produced by DGE were 3890, 1422 and 1765 and down-regulated were 3251, 2869 and 1954 under the Ck, Cr and Cr + GSH conditions, respectively while ZS 758 was taken as a control and Zheda 622 as a treatment (Fig. 2). Similarly, Additional file 2: Figure S3 indicated that in TPA analysis when compared the Zheda 622 vs ZS 758 then number of up-regulated DEGs were more (7059) as compared to down-regulated (4876). Dissimilarly, in reciprocal comparison i.e. ZS 758 vs Zheda 622 up-regulated DEGs became less (4876) than the down-regulated (7059), while in both comparisons if one cultivar was taken as a control then the other was taken as a treatment. Moreover, data stated that Cr significantly reduced the number of up-regulated DEGs as compared with the untreated control. Exogenously applied GSH significantly increased number of up-regulated as well as reduced the down-regulated DEGs.

**Fig. 5:**
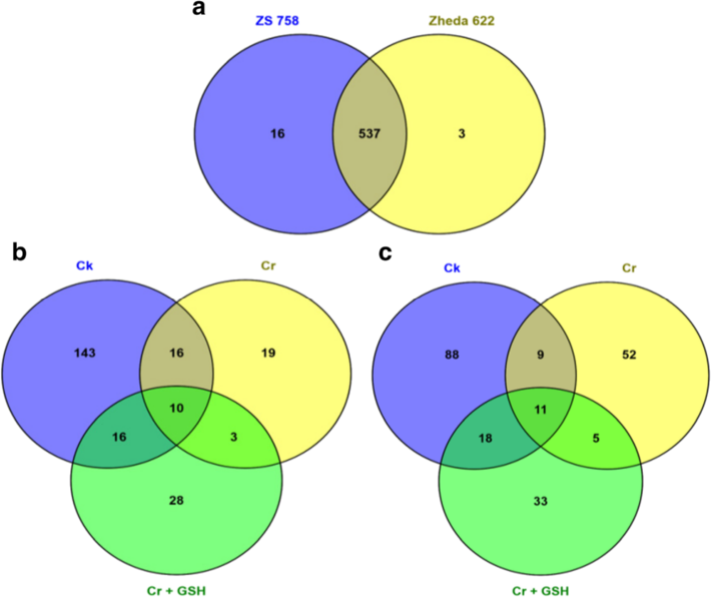
Venny 2.0.2 shows the stress responsive differentially expressed genes (DEGs). Numbers of each circle show the number of stress responsive DEGs that are uniquely (inside of non-overlapping part) or commonly (inside of overlapping part) regulated. Diagram (**a**) shows the stress responsive DEGs between cultivars and total were 556, (**b**) shows the stress responsive DEGs that were up-regulated among treatments i.e. Ck, Cr 400 μM, Cr 400 μM + GSH 1 mM and total were 235, and (**c**) shows the total 216 down-regulated stress responsive DEGs among treatments, were same as mentioned above

Besides, Figs. 3a–h and 4a, b shows the treatments (DGE) and genotype (TPA) specific stress responsive DEGs in both cultivars. There was overlapping between cultivars and 16 stress related genes were included exclusively in ZS 758 as well as 3 were in Zheda 622 (Fig. 5a). Similarly, 143, 19 and 28 genes were found to be up-regulated and 88, 52 and 33 genes became down-regulated that were exclusively in Ck, Cr and Cr + GSH treatments, respectively (Fig. 5b, c). HemI software (Additional file 2: Figure S4) showed the cluster analyses among the Ck, Cr and Cr + GSH treatments (A), respectively and between cultivars (B).

### Gene ontology (GO) functional classification (WEGO) of DEGs and cluster of orthologous groups (COGs) classification

**Fig. 6:**
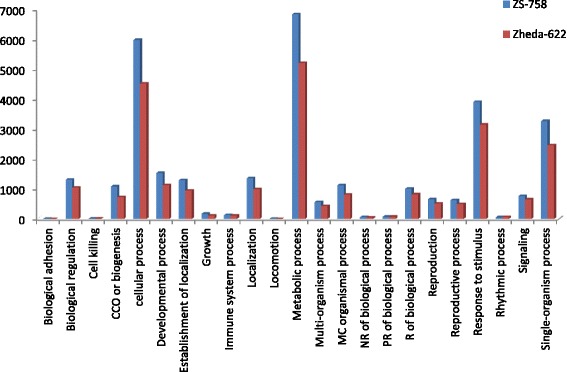
Comparative GO biological regulation between ZS 768 and Zheda 622 under Ck

**Fig. 7:**
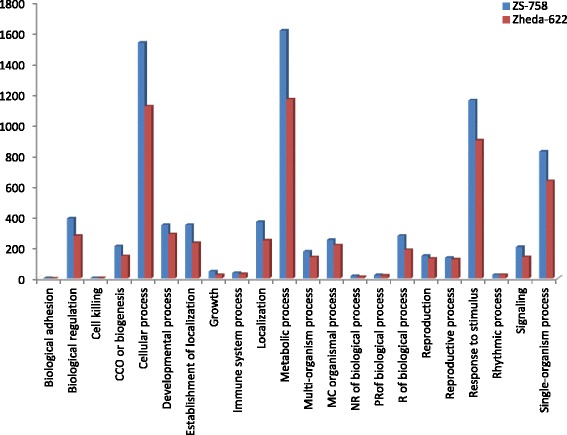
Comparative GO biological regulation between ZS 768 and Zheda 622 under Cr 400 μM treatment condition

**Fig. 8:**
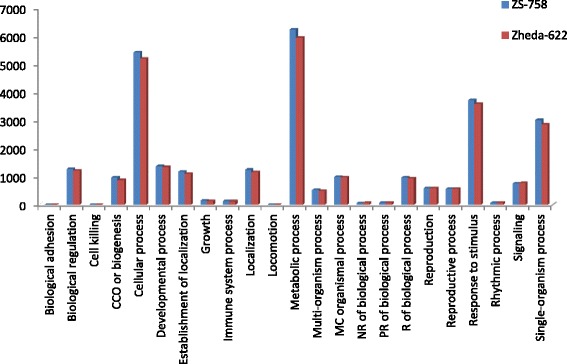
Comparative GO biological regulation between ZS 768 and Zheda 622 under Cr 400 μM + GSH 1 mM treatment condition

**Fig. 9:**
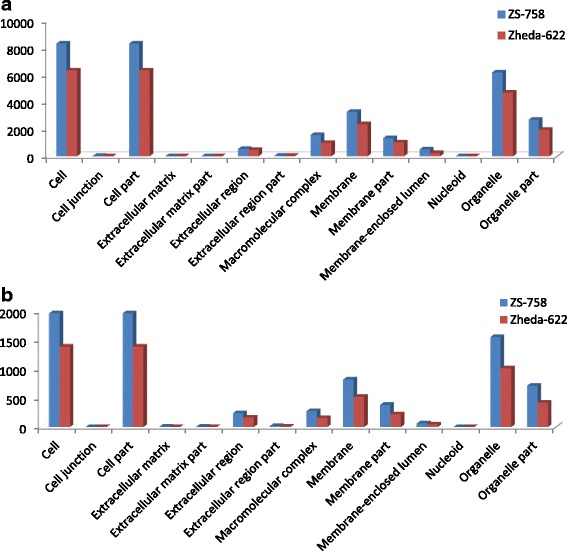
Comparative GO cellular component between ZS 768 and Zheda 622 under Ck (**a**) and Cr 400 μM (**b**), respectively

**Fig. 10:**
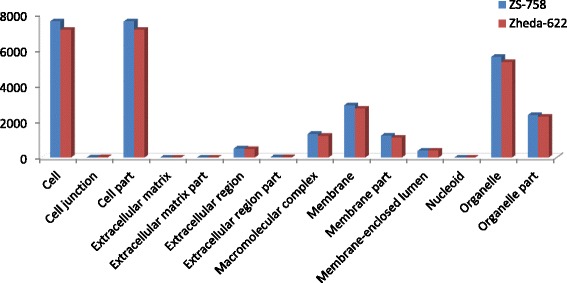
Comparative GO cellular component between ZS 768 and Zheda 622 under Cr 400 μM + GSH 1 mM environmental condition

**Fig. 11:**
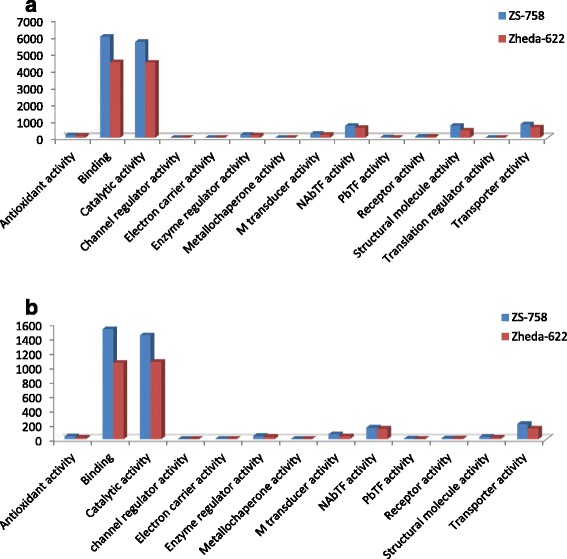
Comparative GO molecular function between ZS 768 and Zheda 622 under Ck (**a**) and Cr 400 μM (**b**), respectively

**Fig. 12:**
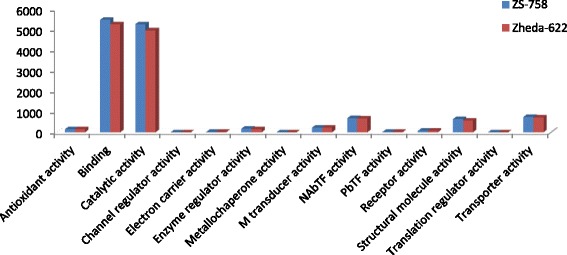
Comparative GO molecular function between ZS 768 and Zheda 622 under Cr 400 μM + GSH 1 mM

DGE generated WEGO treatment dependent (Ck, Cr and Cr + GSH) results were in the form of three categories of GO domains i.e., biological process, cellular component and molecular function. Aggregative, these domains were further contains 51 [49 (22, 14 and 13) in case of Cr 400 μM] GO terms, in which 23 to biological process, and with cellular component and molecular function were 14 each (Figs. 6, 7, 8, 9, 10, 11 and 12). Data showed that prominent GO terms in ‘biological process’ were metabolic process, cellular process, response to stimulus, single organism response and biological regulation (Figs. 6, 7 and 8). The number of genes coding these terms was 6832, 5980, 3908, 3268 and 1534 in ZS-758 and 5209, 4524, 3149, 2457 and 1131 in Zheda-622, respectively under Ck. Then Cr 400 μM decreased the number of genes related to these GO terms by 1613, 1534, 1160, 827 and 391 in ZS-758 and 1166, 1120, 900, 636 and 277 in Zheda-622, respectively. The number of DEGs coding biological regulation GO terms were 6245, 5426, 3727, 3018, 1277 in ZS-758 and 5951, 5208, 3593, 2855 and 1219 in Zheda-622, respectively under Cr 400 μM + GSH 1 mM. The GO terms related to ‘cellular components’ were cell and cell part organelle and organelle part, membrane and macromolecule complex (Figs. 9 and 10). Similarly, number of transcripts coding these terms were 8336, 6208, 3298 and 1575 in ZS-758 and 6348, 4684, 2369 and 986 in Zheda-622 under Ck, respectively. Under the Cr 400 μM alone treatment, the number of genes was decreased to 1969, 1555, 823 and 274 in ZS-758 and 1396, 1014, 520 and 154 in Zheda 622. The number of genes coding cellular component GO terms were 7619, 5627, 2933 and 1324 in ZS-758 and 7151, 5353, 2735 and 1220 in Zheda 622 when subjected to Cr 400 μM + GSH 1 mM. Molecular function’s related prominent GO terms were binding, catalytic activity, transporter activity, nucleic acid binding transcription factor activity, structural molecular activity, antioxidant activity and enzyme regulation, and electron carrier activity (Figs. 11 and 12). The number of genes coding terms were 6009, 5695, 792, 717, 719, 162 and 182 in ZS 758 and 4493, 4465, 629, 599, 430, 132 and 147 in Zheda 622 under untreated control. Under Cr 400 μM treated control, the number of transcripts were 1528, 1442, 209, 160, 33, 39 and 31 in Zheda 622. Lastly, DEGs number that coding the molecular function GO terms were 5466, 5256, 753, 687, 644, 149 and 178 in ZS-758 and 5247, 4957, 716, 674, 570, 144 and 154 in Zheda 622 under the Cr 400 μM + GSH 1. Interestingly, results showed that difference between two cultivars were increased in GO terms such as cellular component (cell and cell part, organellese and organellese part), biological process (biological regulation, response to stimulus and signaling) and molecular function (antioxidant activity, molecular transducer activity and transporter activity) under the Cr stress as compared with control. This is the clear indication that against the GO data the cultivar ZS 758 performed better and has capability to tolerate under the Cr stress. As a whole, these WEGO results were more in Ck as compared to Cr and Cr + GSH. Interestingly, GSH successfully recovered the negative effects of Cr on the both cultivars but the results were more in the favor of ZS-758. Dissimilarly, in TPA analysis all Us were subjected in order to obtain WEGO terms (Additional file 2: Figure S5). TPA results were in the favor of DGE analysis, because GO terms were almost same to that were obtained from DEGs except protein tag, virion and virion part.

**Fig. 13:**
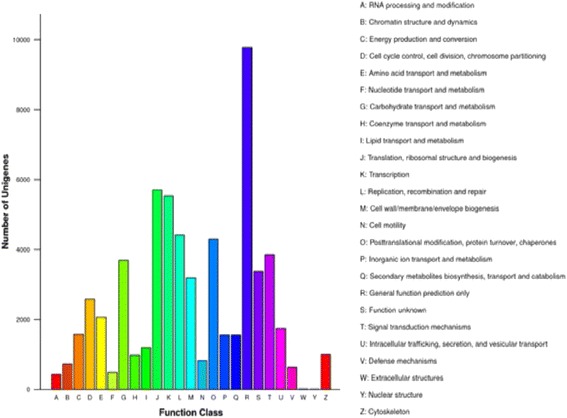
Cluster of orthologous groups (COGs) function classification of all-unigenes (ZS 758 + Zheda 622)

To examine the final gene product (protein), TPA generated all Us were subjected to nr nucleotide data base and calculated the COG classification. Results showed that only 61250 Us were hits to nr data base regarding COG classification with 25 categories (Fig. 13). Among these COG subgroups, most dominant were general function prediction proteins and occupied 9772 genes; others like translation, ribosomal structure and biogenesis (each with 5709); transcription (5540); replication, recombination and repair (each 4420) and post translation modification, protein turnover and chaperone (each 4299). There were also noticed the plant stress respond related COGs such as signal transduction, defense mechanism and co-enzyme transport and metabolism. Contrarily to highest responding COGs, we also noticed the fewest likewise nuclear structures, extracellular structures and RNA processing and modification that were contained only 10, 12 and 430 genes, respectively.

### KEGG metabolic pathway enrichment analysis

**Fig. 14:**
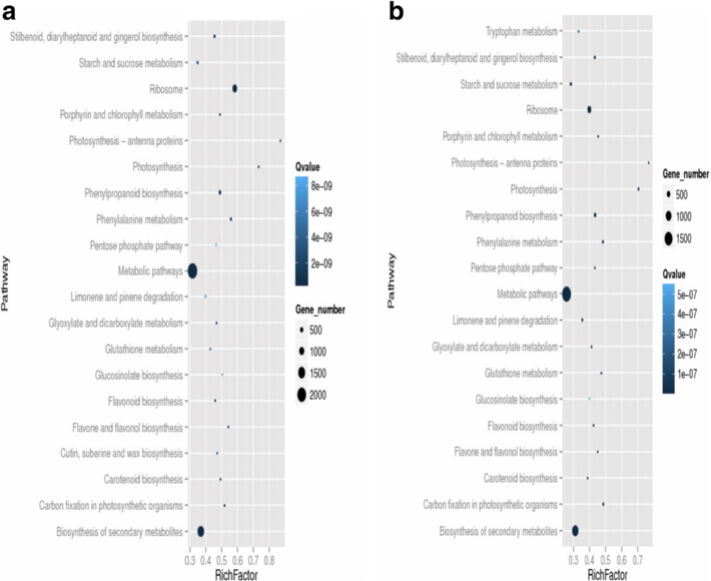
Comparative KEGG pathways analysis between ZS 758 (**a**) and Zheda-622 (**b**) under the Ck

**Fig. 15:**
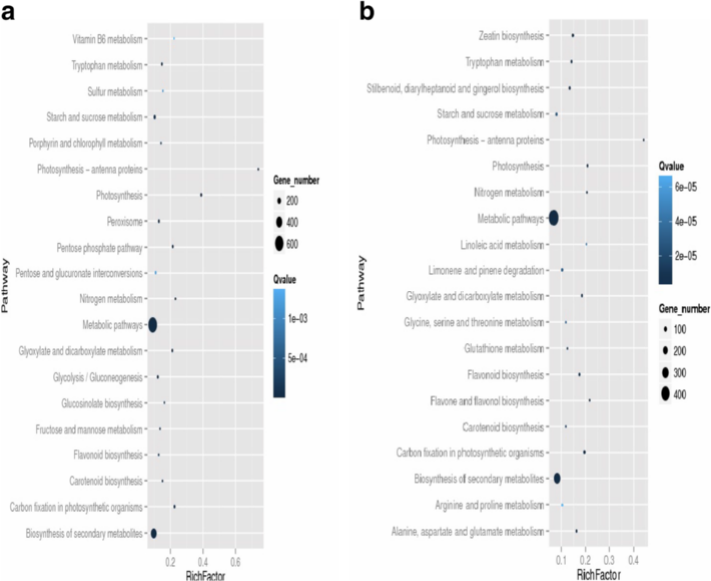
Comparative KEGG pathways analysis between ZS 758 (**a**) and Zheda-622 (**b**) under Cr 400 μM

**Fig. 16:**
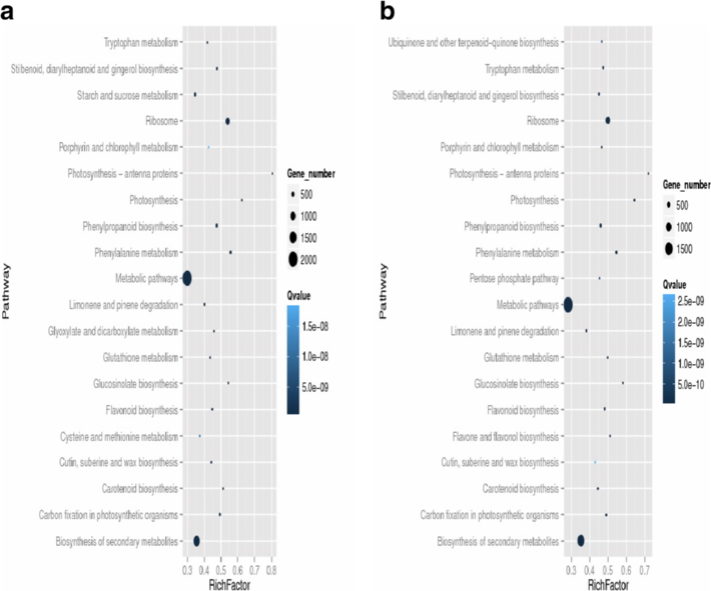
Comparative KEGG pathways analysis between ZS 758 (**a**) and Zheda-622 (**b**) under the Cr 400 μM + GSH 1 mM

Here, DGE generated DEGs were further annotated with Kyoto Encyclopedia of Genes and Genomes (KEGG) database to deep insight the gene product for metabolism and related gene functions in cellular processes. KEGG regarding the DGE data showed the comparative top 20 metabolic pathways under the Ck, Cr and Cr + GSH in ZS 758 (Figs. 14a, 15a and 16a) and Zheda 622 (Fig. 14b, 15b, 16b). Cr stress inhibited the pathways that were related to stilbenoid, diarylhetanoid and gingerol biosyntheisis; phenylpropanoid biosynthesis; phenylalanine metabolism; limonene and pinene degradation; gluthathione metabolism and cutin, suberine and wax biosynthesis in ZS 758 (Fig. 14a) and ribosome; porphyrin and chlorophyll metabolism; pentose phosphate pathway and glucosinolates biosynthesis exclusively in Zheda 622 as compared to Ck (Fig. 14b). Moreover, Cr decreased the rich factor (number of DEGs of the particular pathway/total number of genes mapped to this pathway) in both cultivars for instance starch and sucrose, photosynthesis antenna protein, pentose phosphate pathway, metabolism, carotenoid, carbon fixation in photosynthesis and biosynthesis of secondary metabolites as compared to respective Ck. Interestingly, results also highlighted the cultivars specific KEGG pathways that were induced under Cr stress (15a, 15b). Data showed that Cr induced the vitamin B6, tryptophan biosynthesis, sulphur, peroxisomes, pentose and glucuronate inter-conversions, glycolysis/gluconeogenesis and fructose and manose metabolism in ZS 758. Similarly, in Zheda 622 Cr induced the zeatin biosynthesis, nitrogen metabolism, linoleic acid metabolism, arginine and proline metabolism and alanine, asparate and glutamate metabolism pathways. Moreover, under the same stress, we noticed that glucosinolate pathway was decreased in ZS 758 and inhibited in Zheda 622. As we added the GSH in the solution, it successfully recovered/ improved the KEGG pathways that were inhibited/decreased due to Cr-toxicity in both cultivars (Fig. 16a, b). Furthermore, GSH induced the cysteine and methionine pathway in ZS 758 and ubiquinone and other terpenoid-quinone biosynthesis pathways in Zheda 622. Besides, after the addition of GSH, it increased the rich factor of glucosinolates content related pathway as compared with untreated control but effect was more obvious in Zheda 622 than ZS 758.

Besides DGE, we also performed the KEGG pathway enrichment analysis of all Us by TPA in *B. napus* genotypes (Table 2). Results described the top 20 pathways; prominent pathways were i.e. metabolic pathway, biosynthesis of secondary metabolites, plant pathogen interaction, plant hormonal signal transduction and ribosome. Furthermore, Additional file 1: Table S5 depicts the KEGG results into two levels; level 1 and 2. These two levels were further grouped into five and ten sub-groups, respectively. In level 1 among top 20 pathways, each eight were related to metabolism and genetic information processing; two to cellular process and one each with environmental information processing and organismal systems. Meanwhile, in level 2: five pathways were narrated translation; three related to folding, sorting and degradation; two each with global map, lipid metabolism, nucleotide and transport and catabolism and one each to environmental adaptation, signal transduction, carbohydrate metabolism and biosynthesis of other secondary metabolites.

**Table 2:** TPA generated top 20 KEGG metabolic pathways in cultivar ZS 758 vs Zheda 622, while former treated as a control and later one as a treatment

Sr No.	Pathway	DEGs with pathway annotation (47633) (%)	Level 1	Level 2
1	Metabolic pathways	21	Metabolism	Global map
2	Biosynthesis of secondary metabolites	10.31	Metabolism	Global map
3	Plant-pathogen interaction	5.76	Organismal Systems	Environmental adaptation
4	Plant hormone signal transduction	5.44	Environmental Information Processing	Signal transduction
5	Ribosome	4.77	Genetic Information Processing	Translation
6	RNA transport	4.28	Genetic Information Processing	Translation
7	Spliceosome	4.07	Genetic Information Processing	Transcription
8	Endocytosis	3.43	Cellular Processes	Transport and catabolism
9	Glycerophospholipid metabolism	3	Metabolism	Lipid metabolism
10	Protein processing in endoplasmic reticulum	2.88	Genetic Information Processing	Folding, sorting and degradation
11	Starch and sucrose metabolism	2.50	Metabolism	Carbohydrate metabolism
12	mRNA surveillance pathway	2.31	Genetic Information Processing	Translation
13	Ether lipid metabolism	2.27	Metabolism	Lipid metabolism
14	Ribosome biogenesis in eukaryotes	2.11	Genetic Information Processing	Translation
15	Purine metabolism	1.95	Metabolism	Nucleotide metabolism
16	RNA degradation	1.93	Genetic Information Processing	Folding, sorting and degradation
17	Ubiquitin mediated proteolysis	1.85	Genetic Information Processing	Folding, sorting and degradation
18	Pyrimidine metabolism	1.84	Metabolism	Nucleotide metabolism
19	Phenylpropanoid biosynthesis	1.60	Metabolism	Biosynthesis of other secondary metabolites
20	Phagosome	1.60	Cellular Processes	Transport and catabolism

### Analysis of stress responsive DEGs

#### Cultivar specific stress responsive DEGs

According to results, among 16 genes 12 were related to heat shock protein (HSP), one gene each to molecular chaperone, and absicic acid (ABA) receptor and two were unknown (Table 3). In the same way, among three genes of Zheda 622 each one related to SNF1-related protein kinase catalytic subunit alpha KIN10 (SNF-1RKKIN-10), HSP and chaperone protein dnaJ49. According to ko_definition, mostly genes were responded to stress environment (heat shock 70 kDa protein 1/8 and stress induced phospho-pritein-1). Moreover, presence of more stress responsive genes in ZS 758 might be the reason of its more tolerant behavior than Zheda 622 against adverse conditions (Cr stress, in this case).

**Table 3:** Unique stress responsive genes that are present in two cultivars ZS 758 and Zheda 622, separately

Sr No.	Gene ID	Log2 value	Nr-annotation	Swissprot-annotation	ko_definition
Genes that are included inclusively in ZS 758					
1	CL10944.Contig1_All	−0.026	heat shock protein 70	Heat shock 70 kDa protein	Heat shock 70 kDa protein 1/8
2	CL1423.Contig10_All	−0.384	F5O11.2	Heat shock protein	Stress-induced-phosphoprotein 1
3	CL1423.Contig11_All	0.491	F5O11.2	Heat shock protein	Stress-induced-phosphoprotein 1
4	CL1423.Contig 3_All	0.899	F5O11.2	Heat shock protein STI	Stress-induced-phosphoprotein 1
5	CL1705.Contig5 _All	1.619	DNAJ heat shock N-terminal domain-	--	--
6	CL5492.Contig4 _All	0.576	Octicosa peptide/Phox/Bem1p domain-containing protein	--	--
7	CL6790.Contig 2_All	1.151	Hypothetical protein	Heat shock 70 kDa protein	Heat shock 70 kDa protein 1/8
8	Unigene23139_All	1.515	Chain C, crystal structure of Pyl10-Hab1 complex in the absence of abscisic acid.	Abscisic acid receptor PYL10	Abscisic acid receptor PYR/PYL family
9	Unigene25344_All	1.627	F5O11.2	Heat shock protein STI	Stress-induced-phosphoprotein 1
10	Unigene31019_All	−1.677	Os03g0277300	Heat shock 70 kDa protein	Heat shock 70 kDa protein 1/8
11	Unigene33288_All	−0.26	TO23-1	Heat shock 70 kDa protein	Heat shock 70 kDa protein 5
12	Unigene33299_All	1.021	Hypothetical protein	Heat shock 70 kDa protein	Heat shock 70 kDa protein 1/8
13	Unigene35522_All	0.332	Heat shock protein 90	Heat shock protein 90-1	Molecular chaperone HtpG
14	Unigene36282_All	0.506	Heat shock protein 70	Chaperone protein dnaK	Molecular chaperone DnaK
15	Unigene36807_All	0.76	Heat shock protein 70, partial	Heat shock 70 kDa protein	Heat shock 70 kDa protein 1/8
16	Unigene39664_All	−0.84	Heat shock protein 70, partial	Heat shock 70 kDa protein	Heat shock 70 kDa protein 1/8
Genes that are included inclusively in Zheda 622					
1	CL4329.Contig7 _All	0.34	SNF1-related protein kinase catalytic subunit alpha KIN10	SNF1-related protein kinase catalytic subunit alpha KIN10	--
2	Unigene43699_All	0.67	DnaK-type molecular chaperone HSP 70	Heat shock cognate 70 kDa protein	Heat shock 70 kDa protein 1/8
3	Unigene43946_All	0.57	DNAJ heat shock N-terminal domain-containing protein	Chaperone protein dnaJ 49	DnaJ homolog subfamily B member 12

#### Treatment dependent stress responsive DEGs

**Fig. 17:**
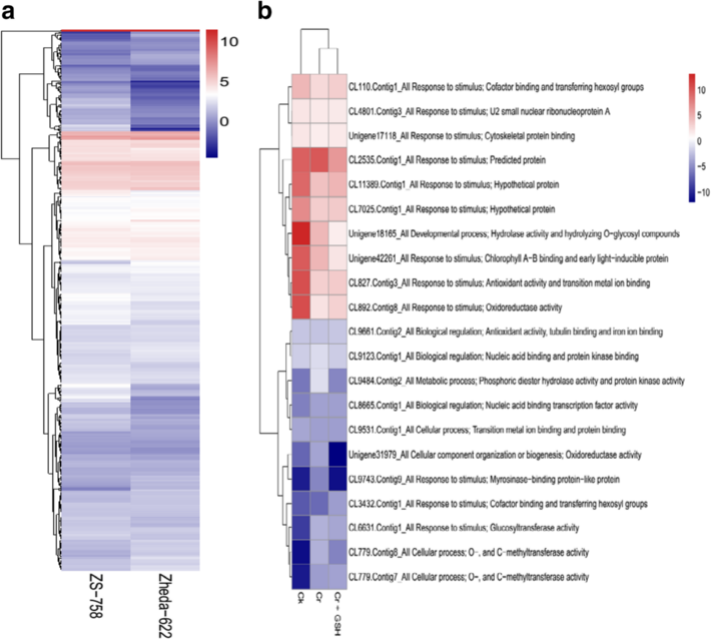
Pheat map (R-package) shows the stress responsive relatively differentially expressed genes between cultivars (ZS 758 and Zheda 622) (**a**), and among treatments i.e. control, Cr 400 μM, Cr 400 μM + GSH 1 mM (**b**)

Similar to cultivars, we analyzed the stress responsive DEG that responds differently under the Ck, Cr and Cr + GSH conditions (Fig. 17b). Pheat map (R-package) showed 21 genes that common among all treatments, were related to stress response. Results indicated that Cr significantly reduced the expression of transcripts were involved in response against stimulation i.e. U_2_ small nuclear protein A, antioxidant activity and transition metal ion binding, cofactor binding and transferring hexosyl groups and cytoskleton protein binding. Besides, Cr also decreased the Log2 ratio of genes related to developmental process i.e. hydrolase activity and hydrolyzing O-glycosyle compounds as compared with Ck. GSH application statistically alleviated the harmful effects of Cr and up-regulated the above mentioned transcripts. Furthermore, we noticed that Cr increased the genes expression of activities that were related to nucleic acid binding transcription factor, O-, and C-methyle transferase, oxidoreductase (that involved in cellular component), phosphoric diester hydrolase and protein kinase, glucosyle transferase and nucleic acid and protein kinase bindings as compared to both Ck and GSH.

#### Transcription factors (TFs)

Results indicated that when DEGs blasted against the TFs, total 10585 genes were hit. Here, Venn diagram showed that 184 TFs were included inclusively in ZS 758 and 86 in Zheda 622 (Additional file 2: Figure S6). On the basis of FPKM value, we selected top 20 TFs in each cultivar. Most prominent TF families in ZS 758 were WRKY, NAC, MYB (6, 5 and 4 times found, respectively) and others were as bZIP, AP2 and TCP each only 1-times found (Table 4). In Zheda 622, dominant TF families were bZIP, NAC, WRKY (6, 6 and 4 times found, respectively) and other like ERF was 2 times, CO-Like and CAMTA each was one time (Table 5). MYB and TCP groups of TF families were not found in Zheda 622 and CAMTA not to ZS 758. Furthermore, according to GO process, in case of ZS 758 most of TFs proteins were related to translation, defense and signal transduction. Similarly, in Zheda 622 most were related to inter membrane transportation, defense and fewer to signal transduction.

**Table 4:** Top 20 transcription factors (TFs) that included inclusively in cultivar ZS 758

Gene ID	FPKM	Nr-Evalue	Nr-annotation	ko_definition	TF-Family
Unigene24917_All	10.6612	6.00E-32	MYB transcription factor BoMyb28-3	MYB proto-oncogene protein, plant	MYB
CL6145.Contig2_All	9.4768	7.00E-60	Hypothetical protein	disease resistance protein RPM1	NAC
Unigene30826_All	8.6974	--	--	--	ERF
CL12987.Contig2_All	8.3208	1.00E-48	Predicted protein)	Small subunit ribosomal protein S26	NAC
Unigene25588_All	8.2822	4.00E-78	Disease resistance protein	--	WRKY
CL10747.Contig2_All	6.8436	4.00E-82	Hypothetical protein	Large subunit ribosomal protein L18A	NAC
Unigene30080_All	6.6148	1.00E-45	Uncharacterized protein	--	ERF
CL13676.Contig1_All	6.5054	9.00E-94	Disease resistance protein	DNA-directed RNA polymerases I, II, and III subunit RPABC1	WRKY
Unigene32296l_All	6.4845	4.00E-64	Hypothetical protein	Small subunit ribosomal protein S7	MYB
Unigene23979_All	5.233	1.00E-76	60S ribosomal protein L23	large subunit ribosomal protein L23	WRKY
Unigene30561_All	4.543	2.00E-17	Putative non-LTR retro-element reverse transcriptase	--	bZIP
CL14102.Contig2_All	4.4415	1.00E-06	Omega gliadin	RNA polymerase II-associated factor 1	AP2
Unigene32262_All	4.1944	1.00E-82	Guanine nucleotide binding protein beta polypeptide 2-like 1 (Partial)	Guanine nucleotide-binding protein subunit beta-2-like 1 protein	MYB
CL9697.Contig6_All	4.0876	7.00E-22	Predicted protein	--	NAC
CL13676.Contig4_All	4.0868	6.00E-177	Disease resistance protein	Disease resistance protein RPM1	WRKY
Unigene32168_All	3.9752	3.00E-08	MYB family transcription factor	--	G2-like
Unigene28076_All	3.9734	1.00E-21	Hypothetical protein	Large subunit ribosomal protein L28	TCP
Unigene31155_All	3.9531	0	Predicted protein	Maintenance of ploidy protein MOB1 (MPS1 binder 1)	WRKY
Unigene32544_All	3.7908	5.00E-118	Conserved unknown protein	Small subunit ribosomal protein S6	NAC
Unigene25175_All	3.6161	4.00E-09	Disease resistance protein-like	Maintenance of ploidy protein MOB1 (MPS1 binder 1)	WRKY

**Table 5:** Top 20 transcription factors (TFs) that included inclusively in cultivar Zheda 622

Gene ID	FPKM	Nr-Evalue	Nr-annotation	ko_definition	TF-Family
Unigene44429_All	9.16	1.00E-09	Expressed protein	Phospholipase D	bZIP
Unigene47751_All	6.81	5.00E-06	Hypothetical protein	Phospholipase D	bZIP
Unigene47805_All	5.18	5.00E-06	Similar to trefoil factor	ATP-binding cassette, subfamily B (MDR/TAP), member 1	NAC
CL11434.Contig2_All	4.37	9.00E-43	Predicted protein	--	WRKY
Unigene44704_All	3.82	3.00E-35	Predicted protein	--	WRKY
Unigene48138_All	3.77	6.00E-32	DNAse I-like superfamily protein	Nucleoporin-like protein 2	bZIP
CL8533.Contig3_All	3.51	3.00E-48	Predicted protein	Histone H4	CO-like
Unigene44558_All	3.36	1.00E-10	Hypothetical protein	Serine/threonine-protein kinase ATR	ERF
Unigene47969_All	3.33	1.00E-19	Protein kinase domain-containing protein	--	WRKY
Unigene47853_All	3.30	4.00E-15	Putative glycosyl hydrolase family 7 protein	Solute carrier family 39 (zinc transporter), member 1/2/3	bZIP
Unigene46084_All	2.85	8.00E-23	Uncharacterized protein	Phospholipase D	bZIP
Unigene43806_All	2.82	2.00E-30	Eukaryotic translation elongation factor 2	Elongation factor 2	NAC
Unigene48179_All	2.77	1.00E-19	40S ribosomal protein S6	Small subunit ribosomal protein S6	NAC
CL6353.Contig1_All	2.76	2.00E-11	Vegetative cell wall protein gp1	Solute carrier family 39 (zinc transporter), member 1/2/3	bZIP
Unigene44057_All	2.65	2.00E-26	Eukaryotic translation elongation factor 2	Elongation factor 2	NAC
Unigene43501_All	2.62	1.00E-18	Ty1_Copia-element protein	--	CAMTA
CL1203.Contig4_All	2.61	1.00E-34	MATE efflux family protein	Multidrug resistance protein, MATE family	ERF
Unigene43297_All	2.58	3.00E-24	Eukaryotic translation elongation factor 2	Elongation factor 2	NAC
Unigene45325_All	2.56	5.00E-45	Disease resistance-like protein	--	WRKY
Unigene49025_All	2.5072	4.00E-103	Beta-glucosidase	--	NAC

Besides the TFs produced by TPA analyses we also blasted the DEGs that were generated by DGE analysis in order to further insight the difference between *B. napus* cultivars under the Ck, Cr and Cr + GSH conditions (Additional file 1: Table S4–S9). Results stated that TFs under the Ck (in ZS 758) were related to transition metal ion binding, oxidoreductase activity, hydrolyase activity, phosphatase and pyrophosphatase activity, and antioxidant activity. Moreover, regulatory proteins for instance cation binding, iron ion binding and transferase activity (transferring the phosphorus-containing groups) were also prominent under the untreated control in ZS 758 cultivar. Similarly, Zheda 622 under the same growth conditions expressed the transcripts coding TFs were uniquely related to structural molecular activity, nucleic acid binding transcription factor activity, vitamin binding, ion binding, and beta-glucosidase activity. Furthermore, Zheda 622 expressed the genes that coding TFs regarding the ligase activity, carboxylesterase activity, DNA binding and protein dimerization activity under Ck environment. Data showed that Cr was added into the solution TFs related to hydrolase activity (hydrolyzing O-glycosyl compounds), ion bindings, antioxidant and catalytic activities were up-regulated. Moreover, under the same treatment, regulatory proteins regarding iron ion binding and phosphatase activity were also up-regulated in ZS 758. In the same way, oxidoreductase activity (acting on the CH-NH2 group of donors, oxygen as acceptor) and hydrolase activity [acting on carbon-nitrogen (but not peptide) bonds, in linear amides] were unique to Zheda 622 under the Cr-toxicity. Results showed that exogenously applied GSH recovered the TFs in ZS 758 that were related to transition metal ion binding; oxidoreductase activity, carboxy-lyase activity, nucleic acid binding transcription factor activity. Moreover GSH also induced nuclear proteins such as hydrolase activity, acting on ester bonds, protein kinase activity, signal transducer activity, oxidoreductase activity (acting on the aldehyde or oxo group of donors, NAD or NADP as acceptor), identical protein binding, structure-specific DNA binding, nucleoside-triphosphatase activity, translation factor activity, nucleic acid binding, guanyl ribonucleotide binding cofactor binding, glutathione disulfide oxidoreductase activity, aminoacyl-tRNA ligase activity and beta-glucosidase activity in ZS 758. Similarly, GSH induced the TFs in Zheda 622 that related to cofactor binding, cytoskeletal protein binding and RNA binding.

#### Protein-protein interactions

**Fig. 18:**
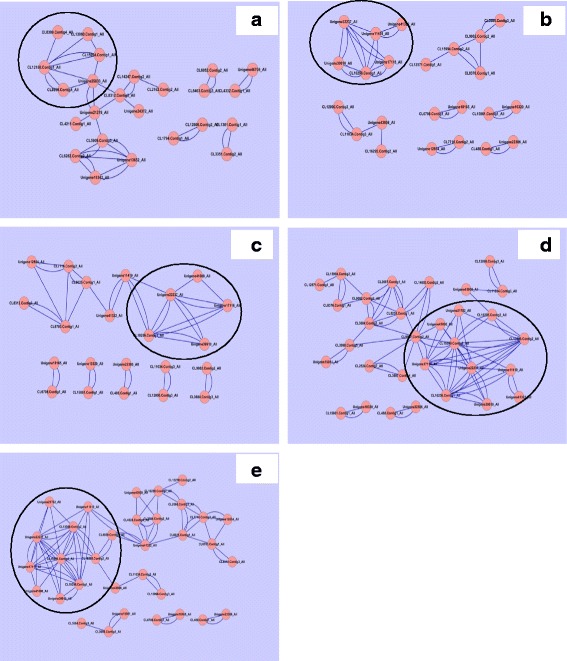
Pro2proNet0.99 represents the protein-protein interactions between cultivars and among treatments. Diagram (**a**) shows ZS 758, (**b**) Zheda 622, (**c**) control, (**d**) Cr 400 μM, and (**e**) Cr 400 μM + GSH 1 mM. Among treatments, cultivar ZS 758 takes as control, while cultivar Zheda 622 as treatment

On the basis of genes expression correlations (more than 0.85) data, total number of Us and CLs were blasted against protein data base and figured out the five and four protein-protein interactions in ZS 758 and Zheda 622, respectively (Fig. 18a, b). In ZS 758, promising interactions were; gene CL-12150 responds to metal ion binding made the correlations with structural molecular activity (CL-8399 and CL-15994), hydrolyase activity (CL-13060), cytoplasmic vesicle (CL-8859) and mitochondrial respiratory chain (U-25833) and Similarly, mitochondrial respiratory chain (MRC) coding protein with structural molecular activity (SMA), metal ion binding (MIB), cytoplasmic vesicle (CV), and RNA and nucleotide binding (RNAnb) (CL-8312). On the other hand, in Zheda 622 among four correlations, two protein’s correlations were with the GO descriptions but other two were unknown. According to Fig. 17b, the first known transcript that was related to intracellular membrane bounded organelles (ICMBO) (CL-10256) made interactions with translation initiation (CL-39910), cytoskeleton protein binding (CBP) and response to stress (CL-39910), and two unknown transcripts such as U-22237 and U-11419; Secondly, SMA coding gene to alkaloids biosynthetic process (CL-12571) and two unknown genes (CL-8370 and CL-9082).

Besides the protein-protein interactions between cultivars, we also performed the same analysis among treatments (CK, Cr and Cr + GSH) powered by DGE approach (Fig. 12c–e). Under the control conditions, we had found three interesting interactions (Fig. 18c). Firstly, U-22237 gene made correlations with nitrile hydratase activity (NHA) (U-41000), CPB and stress response, translation initiation, ICMBO and U-11419. Secondly, iron ion binding protein (CL-12571) interacted with thylakoid protein targeting (CL-8025), transport and hyper-osmotic salinity response (CL-7716), oxidoreductase activity (U-12854) and RNAnb. Lastly, protein coding ICMBO made up the interactions with other proteins i.e. translation initiation, CPB and stress response, U-22237 and U-11419. Cr unveiled the five major protein correlations (Fig. 18d). Prime ones were i.e. CL-15599 (unknown Go description) made interactions NHA, U-21782, CL-16295 (unknown), CL-13306 (unknown), U-11419, translation initiation, U-22237, ICMBO, CPB and response to stress, and CL-9907 (unknown); the gene U-22237 with CL-13306, U-11419, translation initiation, ICMBO, CL-15599 and CL-21782; The transcript CL-9907 tied up with U-41000, CPB and response to stress, CL-15599, hydrolase activity (CL-3887), hydrolyzing O-glycosyle compounds (CL-2524), ICMBO (CL-3098), translation factor activity, NAB (CL-2687) and proteolysis (CL-14803).

When GSH added in Cr solution, it caused the major changes in protein interaction combinations (Fig. 18e) such as in CL-15599 and CL-9907 combinations both were down regulated; in U-22237 correlation, NHA was added and in CL-13306 combination, U-11419 was added as compared than Cr alone treatment. Besides, we also had found five interactions that were up-regulated by GSH as compared than Cr alone. These combinations were; transportation coding protein (CL-7716) made interactions with RNA binding and polymerase activity (CL-2566), oxidoreductase activity (U-12854), Iron binding and thylakoid protein targeting; Kinase activity (CL16288) with signaling and cellular process (CL-4924), CL-2794, RNA binding and polymerase activity, and cation binding, oxido-reductase activity (CL-13276); carotenoid metabolic process (CL-41322) with carboxy-lyase activity (CL-4029), CL-4924, CL-2794 and CL-8025; Lastly thylakoid targeting protein with carotenoid metabolic process, RNA binding and polymerase activity, transport, hyper-osmotic salinity response and iron bindings. Moreover, results stated that by addition of GSH the genes that are responsible for stress environment, signal transduction, transporters, photosynthesis process and RNA polymerization were tied-up together that is the clear indication of plant made shield against Cr stress.

## Discussion

Recent studies evidenced that Cr has wide spectrum of toxicity and causes hazardous effects to many plant species [40]. In current investigation, decreased in dry matter of both cultivars (Table 1) might be due to that Cr negatively regulated the photosystem II, so it reduced the photosynthetic attributes and ultimately plant biomass [41, 42]. Furthermore, metal enrichment in plant cells from solution causes the nutrient imbalance, damages the thylakoid membranes and disruption of mitochondrion structure [10]. Addition of GSH into solution recovered the Cr damages and improved the plant biomass by up-regulating the plant defense machinery (GSH contents itself also) as well as the reduction in metal uptake. Recently, GSH-ascorbate cycle is well known to combat the super oxides (H_2_O_2_, OH^−^ and O_2_^−^), by doing so it deteriorates the metal stress level on plant metabolism [43].

Based on the *de novo* assembly powered by DGE and TPA approaches, our exploration of the genome wide transcriptome profiles in *B. napus* (ZS 758 and Zheda 622) had showed the gene expression pattern and made the new insights available. Moreover, we further investigated the large number of genes that expressed only when GSH came into the solution as compared than Cr alone. This might be due to that GSH enrichment played a triggering role to up-regulate the genes that were down regulated under the Cr alone stress. Cr significantly reduced the number of DEGs as compared to untreated control. Exogenous application of GSH recovered the level of DEGs as compared to Cr alone but that was not equal to Ck. It indicated that *Brassica* plants were still under the stress environment. So, we can assumed that Cr + GSH treatment might induce direct stress responsive genes as well as related to hormonal signal transduction, antioxidant and enzyme regulation.

GO and COG analyses of DEGs further cleared the picture of *B. napus* genome under the Ck, Cr and Cr + GSH. The GO results showed behavior of genes that how they response to diverse environment. Results showed that metal decreased the transcripts by almost 1/4th regarding the important cell organelles, biological regulation, metabolic process, response to stimulus and nucleic acid binding transcription factors activity as compared than Ck. Furthermore, Cr stress also decreased the DEGs related to growth, reproduction, signaling and transporter activity. Interestingly, data showed that Cr inhibited the term locomotion that belong to GO biological process (Fig. 6b) and regulation activity that was in the category of GO molecular function (Fig. 8b). Besides, we noticed that negative effects of Cr were more in Zheda 622. From these results, it can be concluded that Cr drastically damaged the *B. napus* cultivars that might be due to the decrease in plant growth and ultimately the low dry weight (Table 1). As we added the GSH into the Cr solution, it successfully recovered the negative effects of Cr on the plants of both cultivars but of course the results were in the favor of ZS 758 (Figs. 7, 10, 12). Interestingly, our data showed that GSH application significantly minimized the differences regarding the GO terms between the cultivars as compared with Cr and Ck treatments. This finding might be the reason that Zheda 622 was affected more under the Cr stress than ZS 758. Taken as a whole,, under Cr stress plant scavenging mechanism was activated and GSH additions further strengthen the *Brassica* coping system against the unfavorable conditions.

KEGG pathway analysis is a useful bioinformatics software that directed the associations of the genes with the organism and environment. In order to unveil the life processes or metabolisms under different treatments in *B. napus* cultivars, we had blasted the DEGs with KEGG data base. Results showed that Cr stress had negatively regulated the important pathways likewise starch and sucrose, photosynthesis antenna proteins, photosynthesis, pentose phosphate pathway, glucosinolate biosynthesis, carbon fixation during photosynthesis and biogenesis of secondary metabolites in both cultivars compared than Ck. Moreover, our results also showed that Cr inhibited the stilbenoid, diarylheptanoid and gingerol biosynthesis, limonene and pinene degradation, glutathione metabolism and cutin, suberine and wax biosynthesis in ZS 758 and ribose glucosinolate biosynthesis pathways in Zheda 622. On the other hand, ZS-758 induced KEGG pathways for instance vitamin B6, tryptophan, sulfur, pentose and glucuranate inter-conversions and nitrogen metabolism. Similarly, zeatin, linoleic acid metabolism, arginine and proline metabolism and alanine, asparate and glutamate metabolism related pathways were exclusively induced in Zheda 622 in order to cope with Cr stress. It is well established that zeatin is a natural cytokinin hormone that directly related to plant growth. Furthermore, plants synthesized the tryptophan from shikimic acid that is building blocks of protein, and many compounds i.e. serotin, niacin and auxin [44, 45]. Previous studies stated that cluster of pigments are located on the thylakoid membranes called antenna complexes that is known to capture the light [46]. Thus, Cr (in this case) may reduce capacity of light absorbance and ultimately slows down the photosynthesis process. Moreover, data also depicted that nitrogen assimilation pathway was down regulated under the metal stress. From the literature, it is unspoken that nitrogen is one of the important members to synthesize the protein and nucleic acid. It is also constituents of many cellular compounds e.g. alkalide, amides, hormones and enzymes [47, 48]. Taken as a whole, KEGG pathway analysis successfully uncovered the reasons that how, where and the genotype specific behavior of heavy metals, specifically Cr hits to disturb the plant metabolism and also causes the geno-toxcity to deteriorate the plant growth. Exogenously applied GSH not only recovered the decline but also inhibited pathways in both cultivars as compared to respective control. Moreover, GSH had additionally induced the cysteine and methionine metabolism pathway in ZS 758 and ubiquinone and other terpenoid-quinone biosynthesis in Zheda 622 as compared with Ck and Cr stress. From the literature, it is cleared that cystine as an amino acid have the unique role in formation of proteom, essential biomolecules and defense compounds due to its unique reactive sulfhydryl group under the stress condition [49]. In case of methionine, there is well documented that a methionine enzyme named as peptide Met sulfoxide reductase that acts as last-chance antioxidant and played an important role in repairing of proteins that damaged by the oxidative stress [50]. Thus, the inductions of cysteine and methionine may enhance the cell coping capability under the unfavorable conditions. Besides, our data showed that GSH unlikely increased the rich factor of glucosinolate biosynthesis as compared to Ck but interestingly effects was more obvious in Zheda 622 (Fig. 15a–b). Thus we speculated that this novel study along with exploring the Cr mode of action also discovered that how GSH overcomes the metal toxicity when plants are subjected to the stress environment.

Data regarding the cultivar specific stress related DEGs further verified our physiological results that genotypes showed differential response to metal toxicity (Table 3). Results showed that ZS 758 contained DEGs that were related to phospho-protein (that possibly induced by heat stress), molecular chaperone and ABA receptors. Due to the presence of more number of unique stresses related DEGs in ZS 758 as compared than Zheda 622 it was the clear indication that the former cultivar showed the better capability against metal toxicity. From the literature, it is well documented that HSPs act as metal stress protectants and scavenges the negative effects on plants [51, 52]. Recently, Gill et al. [25] confirmed that HSP-90 level was increased under the Cr stress that was in the same line of our RNA sequence analyses results. Previous studies showed that ABA receptors PYRAB-actin resistance1/ PYR-like (PYR1/PYL) draw out the stress respond inside the plant body [53]. Later on, Gonzalez-Guzman et al. [54] stated that same ABA receptors were also responsible for the regulation of stomatal aperture and transcriptional response to ABA. In our results, up-regulation of SNF-1RKKIN-10 might be related to metal toxicity deterioration. Earlier studies evidenced that SNF1-related protein kinase 2 are responsible to degradation of the cadmium stress [55]. Besides, we analyzed common stress responsive genes that were expressed positively in both cultivars under different treatments (Figs. 3 and 4). Our results showed that transcript CL2535.Contig1 was expressed under Cr stress and its expression was increased as compared to untreated control and GSH as well. Both RNA-Seq and qRT-PCR data confirmed that this novel protein (CL2535.Contig1) is responsive to Cr stress. Moreover, data also revealed that theses transcripts i.e. antioxidant activity and transition metal ion binding, cofactor binding and transferring hexosyl groups, hydrolase activity and hydrolyzing O-glycosyl compounds were found to be responsive against GSH as compared with Cr alone. Furthermore, our results (Fig. 3b, Additional file 1: Table S3) also highlighted a novel protein (CL827.Contig3_All) of that expression was increased under the GSH conditions as compared to Cr treated plants.

Since TF (It also known as a sequence specific DNA binding factor) regarded as a protein that uniquely binds with DNA sequences, hence it regulated the level of transcription along with coding information from DNA to mRNA [56, 57]. Furthermore, TF can perform its function individually as well as bind to different proteins in a complex. It also acts as an activator and repressor while recruiting RNA-polymerase enzyme to the relevant transcripts [58]. Results regarding the cultivar specific TFs produced by TPA approach stated that TF families that included inclusively in ZS 758 were WRKY, NAC, MYB, bZIP, AP2 and TCP (Table 4). These families were mainly controlling the function likewise translation, defense and signal transduction in plant body. Similarly in Zheda 622, prominent TF families were the bZIP, NAC, WRKy, ERF, CO-like and CAMTA that controlling inter-membrane transportation, defense and signal transduction (Table 5).

Meanwhile, TF treatment data (that was generated by DGE analysis) suggested that more number of DEGs that blasted TF families were bZIP, trihelix, C2H2, NAC, MYB-related and bHLH in ZS 758 that were mainly related to transition metal ion binding and oxidoreductase activity, hydrolase activity antioxidant activity and iron ion binding, and transferase activity that transferring phosphorus-containing groups. Similarly, in Zheda 622 the most prominent TF families were C2H2, AP2, C3H, bZIP, NAC and ERF that related to structural molecule activity, nucleic acid binding transcription factor activity, DNA binding and protein dimerization activity, ion binding, beta-glucosidase activity and vitamin binding under Ck. As we added Cr into solution, it resulted in the up-regulation of TF families related to hydrolase activity that acting on the glycosyle compounds in ZS 758 (Additional file 1: Table S6) as compared with Ck. Enzymes that catalyze the hydrolysis of the glycosidic linkage of glycosides lead to the formation of a sugar hemiacetal or hemiketal and the corresponding free aglycon. Moreover, hydrolases enzymes are involved in remodeling of cell wall under the stressful regime [59–63]. On the other hand, Cr significantly reduced the TFs that related to antioxidant, catalytic, iron ion binding and phosphatase activities as compared to respective Ck in both cultivars. Furthermore, data (Additional file 1: Table S6 and 7) showed that genes coding TFs that related to the plant defense for instance antioxidant activity, hydrolase activity, transition metal ion binding and iron ion bindings were more expressed in ZS 758 as compared with Zheda 622 under the Cr toxicity. Thus, results clearly indicated that ZS 758 had more tolerance genome as compare with Zheda 622. According to results, GSH significantly recovered the negative effects of Cr on TFs related to transition metal ion binding, oxidoreducatse and carboxylase activities and nucleic acid binding TF activity in both cultivars but the data was in the favor of ZS 758 (Additional file 1: Table S8 and S9). Previous studies suggested that low-iron-inducible ferric chelate reductase responsible for reduction of iron at the root surface. It is likely to be the major Fe (III) chelate reductase in *Arabidopsis* iron metabolism. Steady state mRNA levels are regulated by several metals and its transcription is regulated by FIT1 [64, 65]. The carboxylases such as phosphoenolpyruvate carboxylase have a distinct role during photosynthetic isotope exchange and stomatal conductance in C4 plants [66]. So, we can concluded that the reduction of TF families that related to oxidoreductase and carboxylase activities might be the reason of the deterioration of physio-genomic attributes under the Cr toxicity. The exogenously applied GSH induced the protein kinase and signal transducer activities and oxidoreductase activity that acting on the aldehyde or oxo group of donors, NADP as acceptor. GSH also increased the expression of genes coding TFs regarding triphosphatase activity, translation factor activity and guanyl binding in both cultivars as compared with Cr and Ck. Besides, data also showed the exclusive or cultivar specific role of artificially applied GSH for instance, it induced the glutathione disulfide oxidoreductase activity and amino acyl-tRNA ligase activity that was related to ERF and SBP TF families. Moreover it increased the beta-glucosidase activity that related to FAR1, NAC and C2H2 TF families in ZS-758 (Additional file 1: Table S9). On the other hand, GSH up-regulated the TFs related to oxidoreducatse activity that acting on paired donors with incorporation of molecular oxygen, NAD(P)H as a donor, and incorporation of one atom of oxygen in Zheda 622. Moreover, genes coding TFs such as DNA-binding, protein dimerization activity and RNA binding were also exclusively induced by GSH in Zheda 622. Thus, TFs results further verified our findings that ZS 758 proved to be more tolerant cultivar (Additional file 1: Table S4–S9).

Comprehensive proteomic analyses between cultivars and among treatments were explored (Fig. 7a–e). Results about cultivars showed that MIB protein made interaction with different proteins such as SMA, HA that catalyzed the hydrolysis process, CPVs that involved in metabolism and transportation, and MRC that related to oxidative phosphorylation (Fig. 7a). The oxidative phosphorylation protein coding genes are triggering force to generate the energy rich compound called adenosine triphosphate (ATP) by using oxygen and simple sugar [67, 68]. Similarly, MRC protein interacted with SMA, MIB, CPV, and RNA and nucleotide binding coding proteins. These two protein-protein interactions uncovered the overall cell material transportation system and energy producing mechanism in ZS 758 plant. Furthermore, results explored RNA and nucleotide binding interaction setup that made correlation with MRC, guanyle nucleotide binding, nucleic acid binding and transportation related genes. Regulation of ion transport mechanism is interacted with SPX domain harboring protein, and phosphatase activity and response to stress. It is unspoken that CPB proteins are involved in numerous cell functions such as; it maintains the cell shape, delays cell deformation, helps in cell migration and organelles and vesicles movement in cell [69, 70]. Furthermore, it plays an important role in cell signalling pathways and endocytosis [71], which is involved in chromosomal segregation [69] and cytokinesis [72]. This interaction explored the connection mechanism among the cell organelles such as mitochondria, chloroplast and nucleus bodies. In same genotype, interestingly SMA, CPB and IMBO related proteins together made correlation with unknown gene.

Results showed that under the Ck treatment different proteins such as thylakoid targeting protein, transport, hyper-osmotic salinity response, oxidoreductase activity, and RNA and nucleotide bindings, all together made interaction with iron ion binding protein. From this interaction we could conclude that plant iron ion binding protein may regulate the transportation of elements among cell organelles, improve the structures of thylakoid membrane and RNA and nucleotide binding protein, increase the redox activity, and also it decreased the Na level in cell. Iron-sulfur protein involves in multiple functions such as oxidation-reduction of mitochondrial electron transport and phosphorylation. It also play an important role in SAM-dependent enzymes, biosynthesis of biotin and lipoic acid. Moreover, it further regulates the gene expression [73]. Our results highlighted the ICMBO (such as mitochondria, lysosome, endoplasmic reticulum (ER), Golgi apparatus and endosome) proteins that correlated to translation initiation and CPB and stress response. Thus, ICMBO proteins are helpful to generate the ATP as energy molecule (mitochondria), it take parts in autophagy, phogocytosis and programmed cell death (lysosomes). Furthermore, ICMBO proteins help in folding and also provide the sugar molecules and some other molecules to complex proteins while it attached to nuclear surface (ER) [74]. Thus our data may suggest that the combined function of these proteins were to improve the plant transportation system, enhance the different proteins production, activate the plant defense mechanism, break down or scavenge the metal toxic elements and strengthen the co-ordination among different organelles within the cell to cope the Cr stress. Lorkovic [75] had described that RNA binding proteins are regulators of developmental process, stress response and post transcriptional gene regulations such as pre-mRNA splicing, polyadenylation, RNA stability and RNA export, and chromatin modification. Exogenously applied GSH activated the different proteins that related to defense response and kinase activity, signalling, NA binding and polymerase activity, carotenoid biosynthetic process, and response to metal ion (Cd). This altogether, reduced glutathione activity made ready the *Brassica* plants against the Cr toxicity by speeding-up the transcription process that ultimately leads to more different kinds of coping cell proteins, activated the primary as well as the secondary defense mechanisms, and signal transduction system as compared to Cr alone treatment.

## Conclusion

On the basis of our previous studies that were mainly focused on physiological and biochemical attributes, we applied the DGE and TPA analyses in the present study. This study explored the behavior of Cr-toxicity as well as GSH mode of recovery. Our results showed that Cr up-regulated the several number of stress responsive DEGs, related metabolic pathways like the tryptophan, vitamin B6 sulphur and nitrogen in cultivar ZS 758 and zeatin biosynthesis in cultivar Zheda 622. Cr also highlighted the numerous TFs and proteins that were induced under stressful regime. On the other hand, results also pointed out the novel pathways, TFs and proteins that were inhibited under toxic environment. Exogenously applied GSH not only recovered these negative effects but also increased the expression level of related genes, pathways, TFs and proteins in order to creating the protecting shield in *B. napus* plants against the Cr-toxicity. Findings about physiological and molecular levels suggested that cultivar ZS 758 was proved to be more tolerant cultivar. Further, this study could be taken as a bench mark for those researchers who are working in the field of related stress physiology and molecular biology as well.

## Additional files

Additional file 1: Table S1. DGE generated sequence details (%) under different treatments of chromium (Cr) and reduced glutathione (GSH) in two cultivars of *Brassica napus.* **Table S2** TPA generated sequence read assembly and number of contigs and unigenes in two cultivars of *Brassica napus* (ZS 758 and Zheda 622). **Table S3** Oligonucleotide sequences used in RT-PCR analysis. **Table S4** Top 30 comparative transcription factors (TFs) (selected on the basis of RPKM value) under the control between ZS 758 and Zheda 622. While ZS 758 taken as a standard. **Table S5** Top 30 comparative transcription factors (TFs) (selected on the basis of RPKM value) under the control in ZS 758 and Zheda 622. While Zheda 622 taken as a standard. **Table S6** Top 30 comparative transcription factors (TFs) (selected on the basis of RPKM value) under the Cr 400 μM between ZS758 and Zheda 622. While ZS 758 taken as a standard. **Table S7** Top 30 comparative transcription factors (TFs) (selected on the basis of RPKM value) under the Cr 400 μM between ZS 758 and Zheda 622. While Zheda 622 taken as a standard. **Table S8** Top 30 comparative transcription factors (TFs) (selected on the basis of RPKM value) under the Cr 400 μM + GSH 1 mM between ZS 758 and Zheda 622. While ZS 758 taken as a standard. **Table S9** Top 30 comparative transcription factors (TFs) (selected on the basis of RPKM value) under the Cr 400 μM + GSH 1 mM between ZS 758 and Zheda 622. While Zheda 622 taken as a standard. (DOCX 59 kb)
Additional file 2: Figure S1. Pie-chart shows the *E*-value, similarity and species distribution of *Brassica napus* L. to other plant species. **Figure S2** Scattered plots shows the pair wise comparison of differentially expressed genes, ZS 758 considered as a control and Zheda 622 as a treatment under the different concentrations. In Figure, (A) represents the control, (B) as Cr 400 μM, and (C) represents the Cr 400 μM + GSH 1 mM. **Figure S3** Pie-chart (A) shows the number of up-regulated and down-regulated DEGs in ZS 758/Zheda 622 and (B) Zheda 622/ZS 758, respectively. **Figure S4** HemI hierarchal cluster shows stress responsive relatively differentially expressed genes (DEGs). Diagram (A) shows the DEGs among treatments i.e. Ck, Cr 400 μM, and Cr 400 μM + 1 mM GSH and (B) between cultivars such as ZS 758 and Zheda 622. **Figure S5** Shows the comparative gene ontology functional classification (WEGO) by transcriptome profile analysis in ZS 758 vs Zheda 622. Former cultivar ZS 758 was taken as a control while later cultivar Zheda 622 as a treatment. **Figure S6** Diagram showing the transcription factors (TFs) between cultivars and among the treatments. Numbers of each circle show the number of TFs that are uniquely (inside of non overlapping part) or commonly (inside of overlapping part) regulated. (A) diagram shows the TFs between cultivars and (B) shows the TFs among the treatments. (DOCX 1005 kb)

## Abbreviations

A: Adenine
bps: Base pairs
CBP: Cytoskeleton protein binding
cDNA: Complementary DNA
CLs: Contigs
COGs: Cluster of orthologous groups
Cr: Chromium
CV: Cytoplasmic vesicle
DEGs: Differentially expressed genes
DGE: Digital gene expression
DMRT: Duncan’s multiple range test
DPS: Data processing system
FDR: False discovery rate
gDNA: Genomic DNA
GO: Gene ontology
GSH: Reduced glutathione
HA: Hydrolyase activity
ICMBO: Intracellular membrane bounded organelles
KEGG: Kyoto encyclopedia of genes and genomes
MIB: Metal ion binding
MRC: Mitochondrial respiratory chain
NAB: Nucleic acid binding
NHA: Nitrile hydratase activity
nr: Non-redundant
nts: Nucleotides
RNAnb: RNA and nucleotide binding
ROS: Reactive oxygen species
RPKM: Reads per kb per million reads
SMA: Structural molecular activity
TPA: Transcriptome profile analysis
Us: Unigenes
WEGO: Gene ontology functional classification
