# Supplementary material for: Comparative transcriptome profiling of two Brassica napus cultivars under chromium toxicity and its alleviation by reduced glutathione
Source: BMC Genomics. 2016 Nov 7;17:885. doi: 10.1186/s12864-016-3200-6 (PMC5100228; doi:10.1186/s12864-016-3200-6)
Supplement: Additional file 1: Table S1. — DGE generated sequence details (%) under different treatments of chromium (Cr) and reduced glutathione (GSH) in two cultivars of Brassica napus. Table S2 TPA generated sequence read assembly and number of contigs and unigenes in two cultivars of Brassica napus (ZS 758 and Zheda 622). Table S3 Oligonucleotide sequences used in RT-PCR analysis. Table S4 Top 30 comparative transcription factors (TFs) (selected on the basis of RPKM value) under the control between ZS 758 and Zheda 622. While ZS 758 taken as a standard. Table S5 Top 30 comparative transcription factors (TFs) (selected on the basis of RPKM value) under the control in ZS 758 and Zheda 622. While Zheda 622 taken as a standard. Table S6 Top 30 comparative transcription factors (TFs) (selected on the basis of RPKM value) under the Cr 400 μM between ZS758 and Zheda 622. While ZS 758 taken as a standard. Table S7 Top 30 comparative transcription factors (TFs) (selected on the basis of RPKM value) under the Cr 400 μM between ZS 758 and Zheda 622. While Zheda 622 taken as a standard. Table S8 Top 30 comparative transcription factors (TFs) (selected on the basis of RPKM value) under the Cr 400 μM + GSH 1 mM between ZS 758 and Zheda 622. While ZS 758 taken as a standard. Table S9 Top 30 comparative transcription factors (TFs) (selected on the basis of RPKM value) under the Cr 400 μM + GSH 1 mM between ZS 758 and Zheda 622. While Zheda 622 taken as a standard. (DOCX 59 kb) [file 12864_2016_3200_MOESM1_ESM.docx]

**Table S1** DGE generated sequence details (%) under different treatments of chromium (Cr) and reduced glutathione (GSH) in two cultivars of *Brassica napus* (ZS 758 and Zheda 622)*.*

| **Sample ID** | **Total Reads** | **Total Base Pairs** | **Total Mapped Reads** | **Perfect Match** | **<=2bp Mismatch** | **Unique Match** | **Multi-position Match** | **Total Unmapped Reads** |
| --- | --- | --- | --- | --- | --- | --- | --- | --- |
| ZS 758 CK | 11,874,992 | 581,874,608 | 9,212,605(77.58) | 6,247,130(52.61) | 2,965,475(24.97) | 5,325,255(44.84) | 3,887,350(32.74) | 2,662,387(22.42) |
| Cr-400 µM | 10,905,525 | 534,370,725 | 7,987,140(73.24) | 5,396,916(49.49) | 2,590,224(23.75) | 4,763,511(43.68) | 3,223,629(29.56) | 2,918,385(26.76) |
| Cr-400+ GSH 1 mM | 11,759,780 | 576,229,220 | 9,206,908(78.29) | 5,690,025(48.39) | 3,516,883(29.91) | 5,219,805(44.39) | 3,987,103(33.90) | 2,552,872(21.71) |
| Zheda 622 CK | 11,644,499 | 570,580,451 | 9,200,756(79.01) | 5,957,043(51.16) | 3,243,713(27.86) | 5,324,180(45.72) | 3,876,576(33.29) | 2,443,743(20.99) |
| Cr-400 µM | 12,509,943 | 612,987,207 | 9,308,960(74.41) | 5,964,611(47.68) | 3,344,349(26.73) | 5,496,182(43.93) | 3,812,778(30.48) | 3,200,983(25.59) |
| Cr-400+ GSH 1 mM | 12,148,654 | 595,284,046 | 9,728,709(80.08) | 6,948,110(57.19) | 2,780,599(22.89) | 5,580,970(45.94) | 4,147,739(34.14) | 2,419,945(19.92) |

**Table S2** TPA generated sequence read assembly and number of contigs and unigenes in two cultivars of *Brassica napus* (ZS 758 and Zheda 622).

|  | **Sample** | **Total raw**  **reads** | **Total clean reads** | **Total clean nucleotide (nt)** | **Q20 percentage** | **N percentage** | **GC percentage** |  |
| --- | --- | --- | --- | --- | --- | --- | --- | --- |
|  | ZS 758 | 59427042 | 55439326 | 4989539340 | 0.975 | 0 | 0.4514 |  |
|  | Zheda 622 | 55396686 | 52000468 | 4680042120 | 0.9813 | 0 | 0.448 |  |
|  | **Sample** | **Total number** | **Total length (nt)** | **Mean length(nt)** | **N50** | **Total consensus sequences** | **Distinct clusters** | **Distinct singletons** |
| **Contigs** | ZS 758 | 245841 | 65724205 | 267 | 345 | - | - | - |
|  | Zheda 622 | 220271 | 61641100 | 280 | 381 | - | - | - |
| **Unigenes** | ZS 758 | 91620 | 63860985 | 697 | 1051 | 91620 | 45684 | 45936 |
|  | Zheda 622 | 86328 | 63099683 | 731 | 1112 | 86328 | 43474 | 42854 |
|  | All | 109189 | 83218246 | 762 | 1175 | 109189 | 55753 | 53436 |

**Table S3** Oligonucleotide sequences used in qRT-PCR analysis.

| **Sr. No.** | **Gene ID** | **Gene Description** | **Forward** | **Reverse** |
| --- | --- | --- | --- | --- |
| 1 | CL892.Contig8_All | Oxidoreductase activity | CGGCGATGGAAACTCTAA | CACCAAAGGAAGGCTCT |
| 2 | CL827.Contig3_All | Antioxidant activity and transition metal ion binding | CGCAGATAACAAACTCAA | CTAACAGCCTCCCAAGAC |
| 3 | Unigene42261_All | Chlorophyll A-B binding and early light-inducible protein | ACTGACCACTCGCAAGAT | ACCTCAGCCATACATCTC |
| 4 | CL2535.Contig1_All | Predicted protein | CCACGGTAGCAGCCAATC | AGCAAACGCAGGAACAGG |
| 5 | CL11389.Contig1_All | Hypothetical protein | TGTTTGGGTCTCCCTTTA | AGTCTGCTTCTTCCTCCT |
| 6 | CL7025.Contig1_All | Hypothetical protein | TGGTCGTCTTCTTCGTGT | ACGGTGGATGGAGTCTTG |
| 7 | CL110.Contig1_All | Co-factor binding and transferring hexosyl groups | ACTCACCCAACCCTATGC | CACTTCTCCAATGCCTCA |
| 8 | Unigene18165_All | Hydrolase activity and hydrolyzing O-glycosyl compounds | GCTCATTTCACTCACGCTAC | ATTCCGTTGATGGCTCTT |
| 9 | CL5893.Contig1_All | Peroxidase 52 | ATTCTGCTCGTGGGTTTA | CTTGACTAGCCGTTCTCG |
| 10 | Unigene49190_All | Unknown | ATCAGAAAGGGTGACGAA | CTTGTTTGCTTGGGTGGT |
| 11 | Actin | Standard | TTGGGATGGACCAGAAGG | TCAGGAGCAATACGGAGC |

**Table S4** Top 30 comparative transcription factors (TFs) (selected on the basis of RPKM value) under the control between ZS 758

and Zheda 622. While ZS 758 taken as a standard.

| **Gene ID** | **ZS 758** | **Zheda 622** | **Log2 Ratio (Zheda 622/ZS 758)** | **GO Function** | **TF- Family** |
| --- | --- | --- | --- | --- | --- |
| CL16086.Contig1_All | 2615.67 | 971.80 | -1.43 | Transition metal ion binding and oxidoreductase activity | bZIP |
| Unigene2996_All | 2328.96 | 246.50 | -3.24 | - | CPP, bHLH and bZIP |
| Unigene20146_All | 2290.22 | 595.67 | -1.94 | Transition metal ion binding and hydrolyase activity | bHLH |
| CL14043.Contig1_All | 2031.04 | 871.02 | -1.22 | Binding | ARF and MYB-related |
| Unigene8373_All | 1330.72 | 121.22 | -3.46 | Binding | Trihelix |
| Unigene6389_All | 1207.55 | 215.92 | -2.48 | Binding | Trihelix |
| CL7369.Contig2_All | 1189.42 | 69.77 | -4.09 | Phosphatase activity; and pyrophosphatase activity | TALE |
| CL14003.Contig3_All | 1057.42 | 451.45 | -1.23 | Transition metal ion binding; phospholipid binding; identical protein binding; and antioxidant activity | MYB |
| Unigene17566_All | 885.46 | 83.12 | -3.41 | Binding | ARF, MYB-related |
| CL1301.Contig4_All | 869.27 | 316.38 | -1.46 | Hydrolase activity | C2H2, NAC, TCP, FAR1 |
| Unigene30257_All | 868.63 | 276.28 | -1.65 | - | bZIP, C2H2, AP2, NF-YC, C3H, MYB-related, Trihelix, FAR1, WRKY, C3H and Nin-like |
| CL3381.Contig2_All | 856.46 | 426.49 | -1.01 | - | C3H, MYB-related, bZIP, AP2, WRKY, C2H2,GeBP, Trihelix and ZF-HD |
| Unigene6332_All | 831.48 | 303.05 | -1.46 | - | Trihelix |
| CL16086.Contig2_All | 822.46 | 188.50 | -2.13 | Transition metal ion binding and oxidoreductase activity | bZIP |
| Unigene10713_All | 768.32 | 370.80 | -1.05 | Cation binding | GeBP |
| CL8312.Contig2_All | 734.93 | 87.32 | -3.07 | - | bZIP, C2H2, C3H, MYB-related, NF-YC, Nin-like, NAC, Trihelix, AP2, CAMTA, WRKY, FAR1 and SBP |
| Unigene1251_All | 696.38 | 129.52 | -2.43 | Antioxidant activity; iron ion binding | MYB-related |
| CL10596.Contig3_All | 677.01 | 134.47 | -2.33 | Transition metal ion binding | bZIP |
| CL1301.Contig3_All | 626.14 | 168.06 | -1.90 | Hydrolase activity | C2H2, NAC, TCP, FAR1 |
| Unigene4015_All | 612.28 | 237.96 | -1.36 | Cation binding and hydrolase activity | NAC, C2H2 and TCP |
| CL14674.Contig2_All | 603.73 | 112.16 | -2.43 | Transition metal ion binding and hydro-lyase activity | bHLH |
| Unigene11112_All | 602.41 | 162.63 | -1.89 | Peptide binding; transition metal ion binding; peroxidase activity; and transferase activity | CAMTA |
| Unigene1650_All | 595.66 | 102.27 | -2.54 | Transition metal ion binding; and oxidoreductase activity | bZIP |
| Unigene19469_All | 582.94 | 255.35 | -1.19 | - | bZIP, ZF-HD, bHLH, Trihelix |
| CL1207.Contig4_All | 573.21 | 1566.82 | 1.45 | - | AP2, MYB, C2H2, bZIP, C3H, SBP, ERF, B3, Nin-like and NAC |
| Unigene20280_All | 567.05 | 39.18 | -3.86 | Transferase activity, transferring phosphorus-containing groups | Trihelix, NAC, B3, E2F/DP, AP2, HB-other and bZIP |
| CL10596.Contig2_All | 557.94 | 211.36 | -1.40 | Transition metal ion binding | bZIP |
| CL1207.Contig1_All | 550.28 | 1684.29 | 1.61 | - | AP2, bZIP, C3H, ERF, MYB, NAC, C2H2,SBP, B3, GRAS, G2-like, WRKY, and Trihelix |
| CL14674.Contig3_All | 479.62 | 172.57 | -1.47 | Transition metal ion binding; and hydro-lyase activity | bHLH |
| CL14043.Contig2_All | 477.88 | 152.09 | -1.65 | Binding | ARF and MYB-related |
| CL16086.Contig1_All | 2615.67 | 971.80 | -1.43 | Transition metal ion binding and oxidoreductase activity | bZIP |

**Table S5** Top 30 comparative transcription factors (TFs) (selected on the basis of RPKM value) under the control in ZS 758 and

Zheda 622. While Zheda 622 taken as a standard.

| **Gene ID** | **Zheda 622** | **ZS 758** | **Log2 Ratio (ZS 758/Zheda 622)** | **GO Function** | **TF- Family** |
| --- | --- | --- | --- | --- | --- |
| CL1207.Contig3_All | 4349.61 | 25.99 | 7.39 | - | bZIP, C3H, AP2, ERF, C2H2, NAC, FAR1, SBP, ZF-HD, Trihelix and MYB |
| Unigene21183_All | 1953.76 | 287.39 | 2.77 | Structural molecule activity | C3H, AP2, bZIP, ERF, C2H2, B3 and NAC |
| CL1207.Contig1_All | 1684.29 | 550.28 | 1.61 | - | AP2, bZIP, C3H, AP2, ERF, MYB, NAC, C2H2, SBP, B3, GRAS, G2-like, WRKY, MYB-related and Trihelix |
| Unigene15615_All | 1675.69 | 6.28 | 8.06 | - | AP2, C3H, bZIP, ERF, C2H2, AP2 and Trihelix |
| CL1207.Contig4_All | 1566.82 | 573.21 | 1.45 | - | AP2, MYB, C2H2, bZIP, AP2, C3H, SBP, ERF, B3, Nin-like and NAC |
| CL1618.Contig3_All | 1541.20 | 118.53 | 3.70 | - | LBD |
| Unigene1825_All | 1241.90 | 393.21 | 1.66 | - | M-type |
| CL1207.Contig2_All | 1194.60 | 386.13 | 1.63 | - | C3H, AP2, bZIP, C2H2, SBP, ERF, MYB, NAC, B3, GRAS, MYB-related, E2F/DP and G2-like |
| CL16086.Contig1_All | 971.80 | 2615.67 | -1.43 | Transition metal ion binding; and oxidoreductase activity | bZIP |
| CL15300.Contig1_All | 917.06 | 375.27 | 1.29 | Nucleic acid binding transcription factor activity | bHLH |
| CL14043.Contig1_All | 871.02 | 2031.04 | -1.22 | Binding | ARF, MYB-related |
| CL6483.Contig2_All | 822.10 | 356.13 | 1.21 | Vitamin binding; transition metal ion binding; and oxidoreductase activity; and hydro-lyase activity | B3 |
| Unigene41322_All | 694.83 | 83.28 | 3.06 | - | bZIP, C2H2, NAC, AP2, C3H, Nin-like, SBP, NF-YC, MYB and CAMTA |
| Unigene20146_All | 595.67 | 2290.22 | -1.94 | Transition metal ion binding; and hydro-lyase activity | bHLH |
| Unigene12779_All | 557.55 | 3.83 | 7.18 | - | C2H2, C3H, NAC, C2H2, bZIP, ZF-HD, AP2, WRKY, MYB-related and B3 |
| CL5061.Contig1_All | 547.69 | 191.83 | 1.51 | Ion binding; beta-glucosidase activity | ERF, BES1, Dof and NAC |
| CL15736.Contig1_All | 524.88 | 1.46 | 8.49 | - | AP2, bZIP, C3H, C2H2 and FAR1 |
| CL8312.Contig3_All | 481.36 | 33.42 | 3.85 | - | Trihelix |
| Unigene2510_All | 480.25 | 234.32 | 1.04 | Transition metal ion binding; and structure-specific DNA binding. | GeBP, G2-like, HSF, Whirly, Trihelix, bZIP, AP2 and NF-YC |
| Unigene47061_All | 469.21 | 211.25 | 1.15 | - | YABBY and bZIP |
| Unigene21028_All | 454.44 | 191.99 | 1.24 | Ligase activity. | G2-like and MYB-related |
| CL14003.Contig3_All | 451.45 | 1057.42 | -1.23 | Transition metal ion binding; phospholipid binding; identical protein binding; and antioxidant activity | MYB |
| Unigene5973_All | 449.51 | 126.74 | 1.83 | - | bZIP, AP2, C3H, E2FP, E2F/DP, C2H2, G2-like, ERF, Trihelix, MYB, SBP, MYB-related, B3 and bHLH |
| CL3381.Contig2_All | 426.49 | 856.46 | -1.01 | - | C3H, MYB-related, bZIP, AP2, WRKY, C2H2, MYB, GeBP, Trihelix and ZF-HD |
| CL401.Contig3_All | 415.46 | 0.14 | 11.53 | Carboxylesterase activity | FAR1 |
| CL16604.Contig1_All | 402.28 | 135.52 | 1.57 | Hydrolase activity; and ion binding | ERF, Dof, BES and NAC |
| CL10963.Contig2_All | 389.97 | 183.30 | 1.09 | Nucleic acid binding transcription factor activity; DNA binding; and protein dimerization activity | bZIP |
| Unigene21021_All | 380.18 | 163.45 | 1.22 | - | YABBY |
| Unigene10713_All | 370.80 | 768.32 | -1.05 | Cation binding | GeBP |
| CL1064.Contig11_All | 321.71 | 83.93 | 1.94 | - | YABBY |

**Table S6** Top 30 comparative transcription factors (TFs) (selected on the basis of RPKM value) under the Cr 400 µM between ZS 758 and Zheda 622. While ZS 758 taken as a standard.

| **Gene ID** | **ZS 758** | **Zheda 622** | **log2 Ratio(Zheda 622/ZS 758)** | **GO Function** | **TF- Family** |
| --- | --- | --- | --- | --- | --- |
| CL1812.Contig2_All | 5649.23 | 2299.27 | -1.30 | - | AP2, bZIP, C3H, NAC, C2H2, WRKY and LSD |
| CL1812.Contig1_All | 4046.17 | 1187.03 | -1.77 | - | AP2, C2H2, bZIP, C2H2, C3H, MYB-related, MYB, SBP, WRKY, G2-like, FAR1 and bZIP |
| CL1812.Contig3_All | 2107.56 | 434.01 | -2.28 | - | AP2, bZIP, C3H, C2H2, NAC, FAR1 and SBP |
| CL1812.Contig4_All | 2103.81 | 508.78 | -2.05 | - | AP2, bZIP, C3H, C2H2, NAC, NF-YB, SBP, FAR1 and ERF |
| CL1812.Contig7_All | 2058.66 | 683.39 | -1.59 | - | AP2, bZIP, C3H, C2H2, FAR1, ERF, NAC, MYB, bHLH, SBP, E2F/DP and MYB-related |
| Unigene42705_All | 1935.97 | 632.41 | -1.61 | - | AP2, bZIP, C3H, C2H2, C2H2, WRKY, C3H, SBP, FAR1, MYB, MYB-related, E2F/DP, Trihelix and GRAS |
| Unigene42817_All | 1509.48 | 408.93 | -1.88 | - | AP2, C3H, bZIP, C2H2, FAR1, E2F/DP, SBP, G2-like, ZF-HD, MYB, MYB-related, NAC, WRKY, B3 and G2-like |
| CL3939.Contig2_All | 1490.97 | 487.48 | -1.61 | - | AP2, C2H2, bZIP, C2H2, MYB, SBP, C3H, Trihelix, NAC, ERF, MYB-related and B3 |
| CL1812.Contig6_All | 1272.97 | 445.64 | -1.51 | - | AP2, C2H2, AP2, MYB, ERF, bZIP, Trihelix, E2F/DP, NAC, SBP, bHLH and C3H |
| CL6747.Contig3_All | 1025.91 | 302.35 | -1.76 | Binding | Trihelix |
| Unigene21788_All | 938.51 | 262.76 | -1.84 | Hydrolase activity, hydrolyzing O-glycosyl compounds and ion binding. | BES1, ERF and Dof |
| CL7531.Contig1_All | 833.23 | 319.29 | -1.38 | - | GATA and CAMTA |
| Unigene5390_All | 829.92 | 155.83 | -2.41 | - | AP2 and C3H. |
| CL15736.Contig2_All | 707.38 | 3355.65 | 2.25 | - | AP2, bZIP, C3H, C2H2 and FAR1. |
| Unigene45982_All | 649.64 | 283.56 | -1.20 | Antioxidant activity; iron ion binding and catalytic activity | M-type, MYB-related and Dof. |
| Unigene17276_All | 600.74 | 227.22 | -1.40 | - | YABBY and LBD. |
| CL8312.Contig2_All | 599.72 | 186.33 | -1.69 | - | bZIP, C2H2, C3H, MYB-related, NF-YC, Nin-like, NAC, Trihelix, AP2, CAMTA, WRKY, FAR1 and SBP |
| CL1812.Contig12_All | 559.97 | 212.91 | -1.40 | - | AP2, bZIP, C2H2 and C3H |
| CL6747.Contig2_All | 528.51 | 174.73 | -1.60 | - | Trihelix |
| Unigene30257_All | 526.79 | 197.45 | -1.42 | - | bZIP, C2H2, AP2, NF-YC, C3H, MYB-related, Trihelix, FAR1, WRKY and Nin-like |
| CL1812.Contig8_All | 495.30 | 166.41 | -1.57 | - | AP2, C2H2, MYB, NAC, bZIP, Nin-like, Trihelix, MYB, ERF and SBP |
| CL4851.Contig4_All | 489.57 | 205.74 | -1.25 | Transition metal ion binding | B3 |
| Unigene25458_All | 478.77 | 103.11 | -2.22 | - | YABBY |
| Unigene13198_All | 448.87 | 148.93 | -1.59 | Iron ion binding; catalytic activity; and antioxidant activity | M-type, MYB-related, Dof, MYB and FAR1 |
| CL3939.Contig1_All | 343.86 | 129.57 | -1.41 | - | AP2 |
| CL1082.Contig7_All | 326.78 | 74.32 | -2.14 | - | Dof |
| CL3192.Contig1_All | 290.85 | 139.63 | -1.06 | - | GATA and CAMTA |
| CL4529.Contig2_All | 290.26 | 20.31 | -3.84 | Hydrolase activity, acting on glycosyl bonds and ion binding | ERF, Dof, BES1 and NAC |
| Unigene13861_All | 274.34 | 104.57 | -1.39 | - | bZIP, AP2, C3H, NAC, C2H2, MYB, SBP, C3H, Trihelix and ERF |
| CL7369.Contig2_All | 272.91 | 55.52 | -2.30 | Phosphatase activity; and pyrophosphatase activity | TALE |

**Table S7** Top 30 comparative transcription factors (TFs) (selected on the basis of RPKM value) under the Cr 400 µM

between ZS 758 and Zheda 622. While Zheda 622 taken as a standard.

| **Gene ID** | **Zheda 622** | **ZS 758** | **Log2 Ratio (Zheda 622/ZS 758)** | **GO Function** | **TF- Family** |
| --- | --- | --- | --- | --- | --- |
| CL15736.Contig2_All | 3355.65 | 707.38 | 2.25 | - | AP2, bZIP, C3H, C2H2 and FAR1 |
| CL1812.Contig2_All | 2299.27 | 5649.23 | -1.30 | - | AP2, bZIP, C3H, NAC, C2H2, WRKY, AP2 and LSD |
| CL1812.Contig1_All | 1187.03 | 4046.17 | -1.77 | - | AP2, C2H2, bZIP, C3H, MYB-related, MYB, SBP, WRKY, NAC, G2-like, FAR1 and bZIP |
| CL1812.Contig7_All | 683.39 | 2058.66 | -1.59 | - | AP2, bZIP, C3H, C2H2, FAR1, ERF, NAC, MYB, bHLH, SBP, E2F/DP and MYB-related |
| CL1064.Contig11_All | 654.92 | 266.81 | 1.30 | - | YABBY |
| Unigene42705_All | 632.41 | 1935.97 | -1.61 | - | AP2, bZIP, C3H, C2H2, WRKY, SBP, FAR1, MYB, MYB-related, E2F/DP, Trihelix, GRAS |
| CL6483.Contig2_All | 517.97 | 241.65 | 1.10 | Vitamin binding; transition metal ion binding; oxidoreductase activity, acting on the CH-NH2 group of donors, oxygen as acceptor; and hydro-lyase activity | B3 |
| CL1812.Contig4_All | 508.78 | 2103.81 | -2.05 | - | AP2, bZIP, C3H, C2H2, NAC, C2H2, NF-YB, C3H, SBP, FAR1 and ERF |
| Unigene20062_All | 490.55 | 219.04 | 1.16 | - | GeBP, B3, bHLH, E2F/DP, B3 and bZIP |
| CL3939.Contig2_All | 487.48 | 1490.97 | -1.61 | - | AP2, C2H2, bZIP, MYB, SBP, C3H, Trihelix, NAC, ERF, MYB-related and B3 |
| CL1812.Contig6_All | 445.64 | 1272.97 | -1.51 | - | AP2, C2H2, AP2, MYB, ERF, bZIP, Trihelix, E2F/DP, NAC, SBP, bHLH, ERF and C3H |
| CL1812.Contig3_All | 434.01 | 2107.56 | -2.28 | - | AP2, bZIP, C3H, C2H2, NAC, FAR1 and SBP |
| Unigene41322_All | 413.14 | 12.82 | 5.01 | - | bZIP, C2H2, NAC, AP2, ND, C3H, Nin-like, SBP, C3H, MYB, and CAMTA |
| Unigene42817_All | 408.93 | 1509.48 | -1.88 | - | AP2, C3H, bZIP, C2H2, FAR1, E2F/DP, SBP, G2-like, ZF-HD, MYB, MYB-related, NAC, WRKY and B3 |
| CL9065.Contig1_All | 384.83 | 166.07 | 1.21 | Hydrolase activity; and ion binding | G2-like |
| CL11823.Contig1_All | 347.71 | 172.72 | 1.01 | Oxidoreductase activity; and iron ion binding | B3, GeBP, bHLH, G2-like, bHLH and E2F/DP. |
| CL3843.Contig1_All | 320.73 | 147.52 | 1.12 | Antioxidant activity; catalytic activity; and iron ion binding | M-type |
| CL7531.Contig1_All | 319.29 | 833.23 | -1.38 | - | GATA and CAMTA. |
| CL6747.Contig3_All | 302.35 | 1025.91 | -1.76 | Binding | Trihelix |
| CL488.Contig1_All | 295.02 | 133.94 | 1.14 | - | bZIP, C2H2, NAC, AP2, MYB-related, C3H, SBP, Nin-like and NF-X1 |
| CL6747.Contig1_All | 287.09 | 56.20 | 2.35 | Binding | Trihelix |
| Unigene45982_All | 283.56 | 649.64 | -1.20 | Antioxidant activity; iron ion binding; and catalytic activity | M-type, MYB-related and Dof |
| CL12992.Contig2_All | 280.45 | 62.54 | 2.16 | - | M-type |
| Unigene21788_All | 262.76 | 938.51 | -1.84 | Hydrolase activity, hydrolyzing O-glycosyl compounds; and ion binding | BES1, ERF and Dof |
| CL12992.Contig1_All | 256.18 | 41.12 | 2.64 | - | M-type |
| CL9279.Contig4_All | 247.49 | 51.09 | 2.28 | - | C3H, C3H, C2H2, NF-YC, GeBP, MYB-related and MYB |
| Unigene28011_All | 243.33 | 73.79 | 1.72 | Transition metal ion binding | bZIP |
| CL2847.Contig6_All | 238.53 | 105.97 | 1.17 | Hydrolase activity, acting on carbon-nitrogen (but not peptide) bonds, in linear amides; transition metal ion binding. | C2H2 |
| Unigene17276_All | 227.22 | 600.74 | -1.40 | - | YABBY and LBD |
| Unigene8507_All | 227.19 | 106.23 | 1.10 | - | Trihelix |

**Table S8** Top 30 comparative transcription factors (TFs) (selected on the basis of RPKM value) under the Cr 400 µM + GSH 1 mM

between ZS 758 and Zheda 622. While ZS 758 taken as a standard.

| **Gene ID** | **ZS 758** | **Zheda 622** | **Log2 Ratio (Zhedea 622/ZS 758)** | **GO Function** | **TF- Family** |
| --- | --- | --- | --- | --- | --- |
| CL9359.Contig2_All | 1373.97 | 575.00 | -1.26 | Transition metal ion binding; oxidoreductase activity; and carboxy-lyase activity | M-type |
| CL1207.Contig1_All | 728.39 | 2254.88 | 1.63 | - | AP2, Bzip, C3H, ERF, MYB, NAC, C2H2, SBP, B3, GRAS, G2-like, WRKY, MYB-related, and Trihelix |
| CL1207.Contig4_All | 584.63 | 1550.37 | 1.41 | - | AP2, MYB, C2H2, bZIP, C3H, SBP, ERF, B3, ERF, Nin-like and NAC |
| CL10596.Contig3_All | 540.19 | 196.74 | -1.46 | Transition metal ion binding | bZIP |
| Unigene2996_All | 524.53 | 258.12 | -1.02 | - | CPP, bHLH and bZIP |
| CL15300.Contig1_All | 496.87 | 162.98 | -1.61 | Nucleic acid binding transcription factor activity | bHLH |
| Unigene16104_All | 485.84 | 242.57 | -1.00 | Hydrolase activity, acting on ester bonds; and nucleic acid binding | bHLH |
| Unigene20146_All | 472.81 | 170.34 | -1.47 | Transition metal ion binding; and hydro-lyase activity | bHLH |
| Unigene25458_All | 464.68 | 63.46 | -2.87 | - | YABBY |
| CL8312.Contig2_All | 453.00 | 115.31 | -1.97 | - | bZIP, C2H2, C3H, MYB-related, NF-YC, Nin-like, Trihelix, AP2, CAMTA, WRKY, FAR1 and SBP |
| Unigene13474_All | 442.98 | 181.23 | -1.29 | Protein kinase activity; signal transducer activity; oxidoreductase activity, acting on the aldehyde or oxo group of donors, NAD or NADP as acceptor; identical protein binding; and structure-specific DNA binding | bZIP, C2H2, bZIP, NAC, C3H, SBP, ERF, AP2, MYB, Nin-like, SBP, NF-YC, and NF-YB |
| Unigene17276_All | 423.96 | 151.77 | -1.48 | - | YABBY and LBD |
| Unigene2673_All | 413.36 | 174.72 | -1.24 | Nucleoside-triphosphatase activity; translation factor activity, nucleic acid binding; and guanyl ribonucleotide binding | WRKY and NAC |
| CL1207.Contig2_All | 404.08 | 1136.40 | 1.49 | - | C3H, AP2, bZIP, C2H2, SBP, ERF, MYB, NAC, B3, GRAS, MYB-related, E2F/DP and G2-like |
| Unigene6332_All | 320.02 | 93.99 | -1.77 | - | Trihelix |
| Unigene28022_All | 280.57 | 89.56 | -1.65 | - | GATA and CAMTA |
| CL1301.Contig3_All | 253.63 | 112.86 | -1.17 | Hydrolase activity, hydrolyzing O-glycosyl compounds; and cation binding | C2H2, NAC, TCP and FAR1 |
| Unigene8373_All | 241.50 | 115.91 | -1.06 | Binding | Trihelix |
| Unigene1827_All | 230.02 | 780.49 | 1.76 | Cofactor binding; and oxidoreductase activity, acting on the CH-OH group of donors, NAD or NADP as acceptor | NAC, ERF and B3 |
| CL8160.Contig1_All | 226.84 | 75.30 | -1.59 | Hydrolase activity | MYB-related |
| CL488.Contig1_All | 222.28 | 615.81 | 1.47 | - | bZIP, C2H2, NAC, AP2, MYB-related, C3H, SBP, Nin-like and NF-X1 |
| Unigene23981_All | 220.93 | 45.22 | -2.29 | Antioxidant activity; catalytic activity; and iron ion binding | M-type, Dof and MYB-related |
| Unigene1745_All | 215.66 | 97.50 | -1.15 | Glutathione disulfide oxidoreductase activity; and aminoacyl-tRNA ligase activity | ERF and SBP |
| CL789.Contig3_All | 210.89 | 99.50 | -1.08 | Binding | ARF and MYB-related |
| Unigene12955_All | 191.86 | 413.73 | 1.11 | Metal ion binding | FAR1, HSF and HB-other |
| CL8162.Contig2_All | 175.31 | 79.99 | -1.13 | Cation binding; and phosphotransferase activity, phosphate group as acceptor | GRAS |
| CL11953.Contig2_All | 172.99 | 47.53 | -1.86 | - | bZIP, AP2, C3H, SBP, C2H2, ERF, MYB-related and ERF |
| CL8994.Contig3_All | 169.53 | 30.17 | -2.49 | Hydrolase activity, acting on ester bonds; protein binding; ion binding; and beta-glucosidase activity | NAC, C2H2 and FAR1 |
| Unigene22386_All | 159.06 | 343.31 | 1.11 | - | bZIP, C3H, Trihelix, AP2, C2H2, G2-like and MYB-related |
| Unigene2822_All | 143.21 | 66.80 | -1.10 | - | GATA |

**Table S9** Top 30 comparative transcription factors (TFs) (selected on the basis of RPKM value) under the Cr 400 µM + GSH 1 mM

between ZS 758 and Zheda 622. While Zheda 622 taken as a standard.

| **Gene ID** | **Zheda 622** | **ZS 758** | **Log2 Ratio (Zheda 622/ZS 758)** | **GO Function** | **TF-Family** |
| --- | --- | --- | --- | --- | --- |
| CL1207.Contig1_All | 2254.88 | 728.39 | 1.63 | - | AP2, bZIP, C3H, ERF, MYB, NAC, C2H2, SBP, B3, GRAS, G2-like, WRKY, MYB-related and Trihelix |
| CL1207.Contig4_All | 1550.37 | 584.63 | 1.41 | - | AP2, MYB, C2H2, bZIP, C3H, SBP, ERF, B3, ERF, Nin-like and NAC |
| Unigene41322_All | 1412.97 | 76.04 | 4.22 | - | bZIP, C2H2, NAC, AP2, C3H, Nin-like, SBP, NF-YC, MYB and CAMTA |
| CL1207.Contig2_All | 1136.40 | 404.08 | 1.49 | - | C3H, AP2, bZIP, C2H2, SBP, ERF, MYB, NAC, B3, GRAS, MYB-related E2F/DP and G2-like |
| Unigene1827_All | 780.49 | 230.02 | 1.76 | Cofactor binding; oxidoreductase activity, acting on the CH-OH group of donors, NAD or NADP as acceptor | NAC, ERF and B3 |
| CL488.Contig1_All | 615.81 | 222.28 | 1.47 | - | bZIP, C2H2, NAC, AP2, MYB-related, C3H, SBP, Nin-like, SBP and NF-X1 |
| CL9359.Contig2_All | 575.00 | 1373.97 | -1.26 | Transition metal ion binding; oxidoreductase activity; carboxy-lyase activity | M-type |
| Unigene12955_All | 413.73 | 191.86 | 1.11 | Metal ion binding | FAR1, HSF and HB-other |
| CL8312.Contig3_All | 382.36 | 9.74 | 5.29 | - | Trihelix |
| Unigene22386_All | 343.31 | 159.06 | 1.11 | - | bZIP, C3H, Trihelix, AP2, C2H2, G2-like and MYB-related |
| CL1937.Contig1_All | 329.45 | 54.49 | 2.60 | - | HB-other, AP2, C3H, NAC and Trihelix |
| CL2240.Contig3_All | 326.43 | 114.74 | 1.51 | Metal ion binding; and cytoskeletal protein binding | C3H |
| CL11403.Contig3_All | 298.44 | 91.44 | 1.71 | Catalytic activity | CAMTA |
| Unigene21850_All | 289.87 | 120.77 | 1.26 | Transferase activity, transferring acyl groups | MYB, ARF and NAC |
| Unigene1573_All | 282.13 | 59.60 | 2.24 | Oxidoreductase activity, acting on paired donors, with incorporation or reduction of molecular oxygen, NAD(P)H as one donor, and incorporation of one atom of oxygen; and iron ion binding. | B3, GeBP, bHLH, G2-like, E2F/DP and bZIP |
| Unigene2996_All | 258.12 | 524.53 | -1.02 | - | CPP, bHLH and bZIP |
| Unigene9659_All | 251.27 | 117.73 | 1.09 | - | NF-YA |
| Unigene16104_All | 242.57 | 485.84 | -1.00 | Hydrolase activity, acting on ester bonds; and nucleic acid binding | bHLH |
| Unigene16290_All | 208.51 | 89.99 | 1.21 | Hydrolase activity, acting on ester bonds | GRAS and NAC |
| CL1744.Contig8_All | 205.85 | 62.91 | 1.71 | - | NF-YC, GeBP and MYB-related |
| Unigene1707_All | 201.91 | 98.48 | 1.04 | Metal ion binding; and endopeptidase activity | HB-other and FAR1 |
| Unigene30370_All | 198.01 | 87.40 | 1.18 | Binding | TALE |
| CL10596.Contig3_All | 196.74 | 540.19 | -1.46 | Transition metal ion binding | bZIP |
| CL13301.Contig2_All | 196.45 | 76.07 | 1.37 | - | NF-YA |
| CL10963.Contig2_All | 194.90 | 54.70 | 1.83 | Nucleic acid binding transcription factor activity; DNA binding; and protein dimerization activity | bZIP |
| Unigene13474_All | 181.23 | 442.98 | -1.29 | Protein kinase activity; signal transducer activity; oxidoreductase activity, acting on the aldehyde or oxo group of donors, NAD or NADP as acceptor; identical protein binding; and structure-specific DNA binding | bZIP, C2H2, NAC, C3H, SBP, ERF, AP2, MYB, Nin-like, NF-YC, NF-YB |
| Unigene2673_All | 174.72 | 413.36 | -1.24 | Nucleoside-triphosphatase activity; translation factor activity, nucleic acid binding; and guanyl ribonucleotide binding | WRKY and NAC |
| Unigene20146_All | 170.34 | 472.81 | -1.47 | Transition metal ion binding; and hydro-lyase activity | bHLH |
| CL15300.Contig1_All | 162.98 | 496.87 | -1.61 | Nucleic acid binding transcription factor activity | bHLH |
| Unigene39910_All | 158.63 | 53.12 | 1.58 | RNA binding | MYB-related |
