# Supplementary material for: Comparative transcriptome profiling of two Brassica napus cultivars under chromium toxicity and its alleviation by reduced glutathione
Source: BMC Genomics. 2016 Nov 7;17:885. doi: 10.1186/s12864-016-3200-6 (PMC5100228; doi:10.1186/s12864-016-3200-6)
Supplement: Additional file 2: Figure S1. — Pie-chart shows the E-value, similarity and species distribution of Brassica napus L. to other plant species. Figure S2 Scattered plots shows the pair wise comparison of differentially expressed genes, ZS 758 considered as a control and Zheda 622 as a treatment under the different concentrations. In Figure, (A) represents the control, (B) as Cr 400 μM, and (C) represents the Cr 400 μM + GSH 1 mM. Figure S3 Pie-chart (A) shows the number of up-regulated and down-regulated DEGs in ZS 758/Zheda 622 and (B) Zheda 622/ZS 758, respectively. Figure S4 HemI hierarchal cluster shows stress responsive relatively differentially expressed genes (DEGs). Diagram (A) shows the DEGs among treatments i.e. Ck, Cr 400 μM, and Cr 400 μM + 1 mM GSH and (B) between cultivars such as ZS 758 and Zheda 622. Figure S5 Shows the comparative gene ontology functional classification (WEGO) by transcriptome profile analysis in ZS 758 vs Zheda 622. Former cultivar ZS 758 was taken as a control while later cultivar Zheda 622 as a treatment. Figure S6 Diagram showing the transcription factors (TFs) between cultivars and among the treatments. Numbers of each circle show the number of TFs that are uniquely (inside of non overlapping part) or commonly (inside of overlapping part) regulated. (A) diagram shows the TFs between cultivars and (B) shows the TFs among the treatments. (DOCX 1005 kb) [file 12864_2016_3200_MOESM2_ESM.docx]

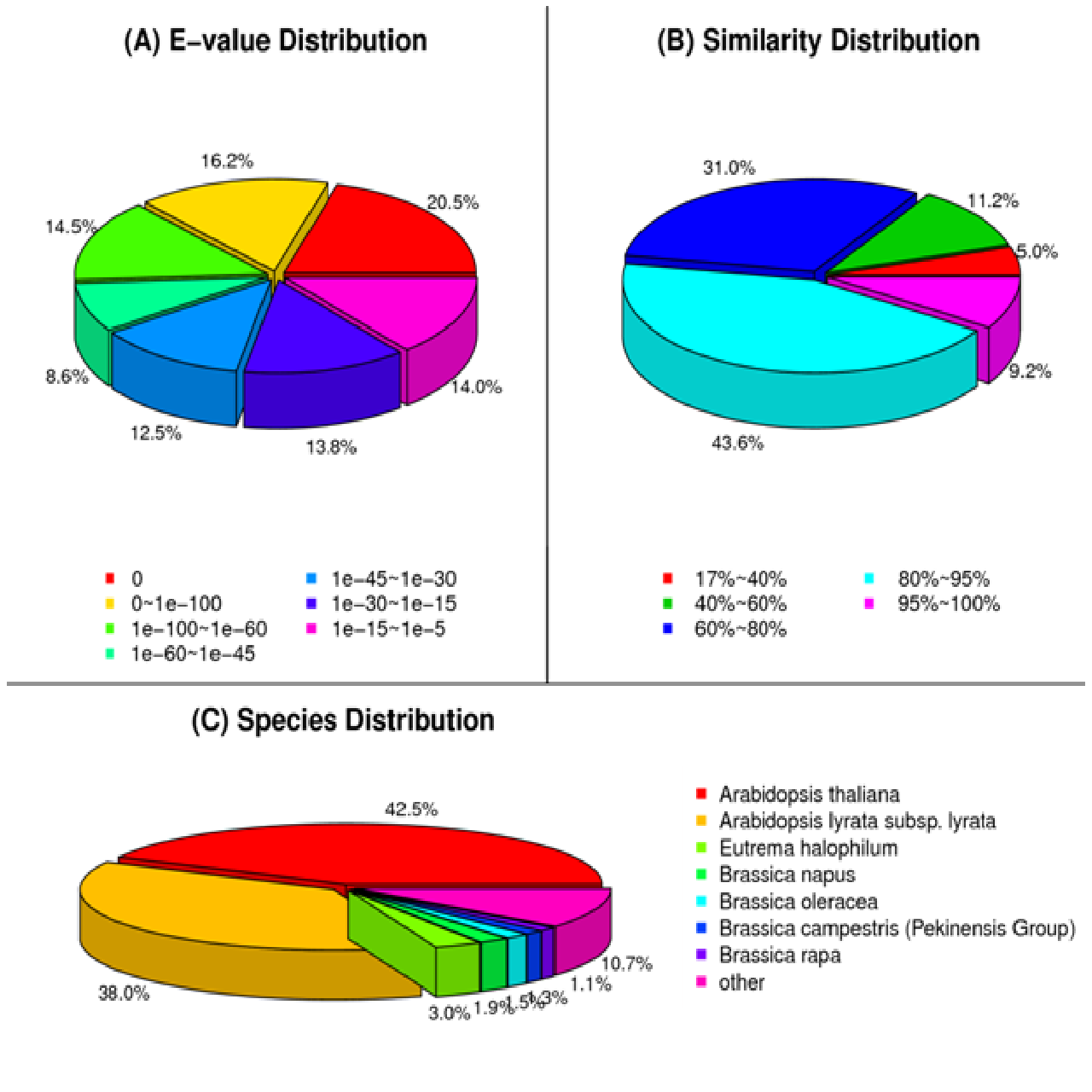


**Fig. S1** Pie-chart shows the E-value, similarity and species distribution of *Brassica napus* L. to other plant species.


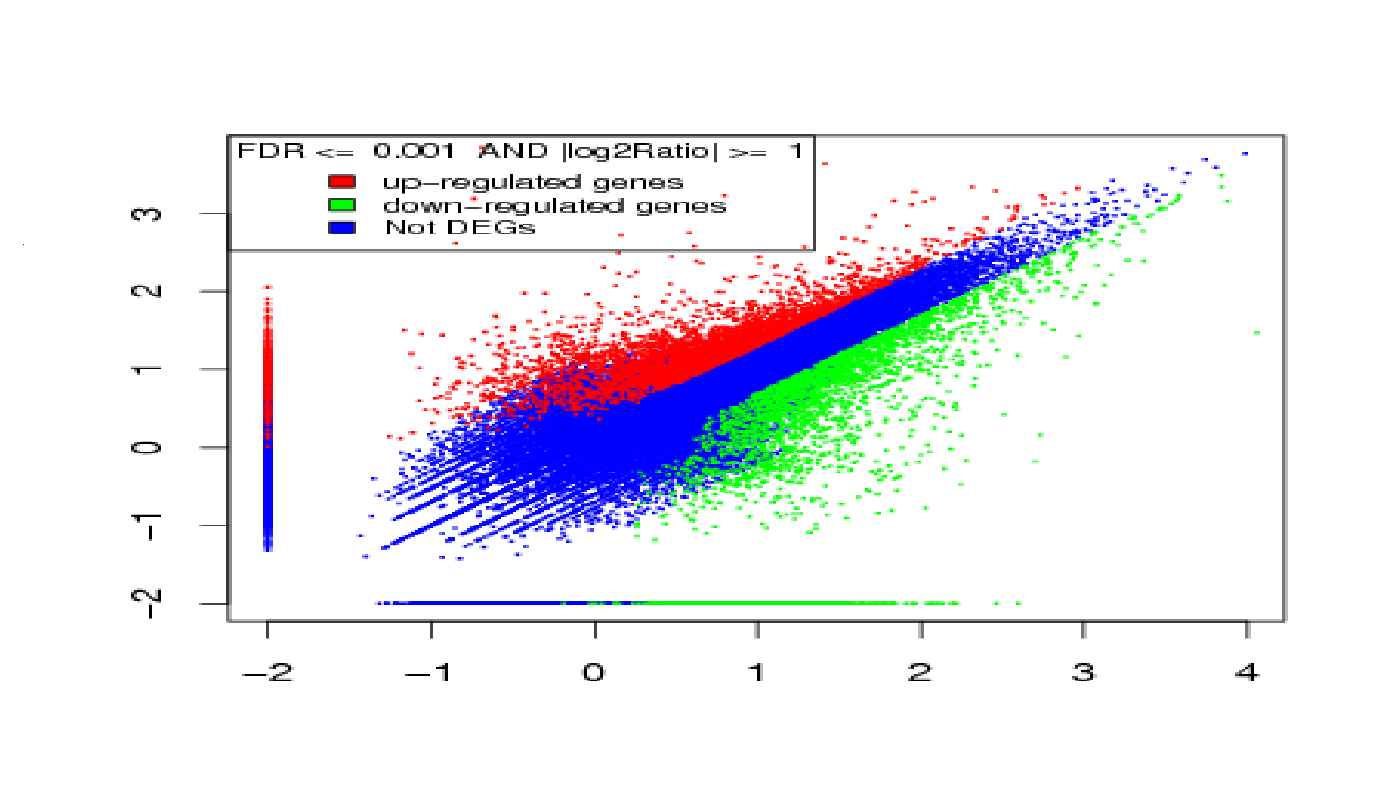

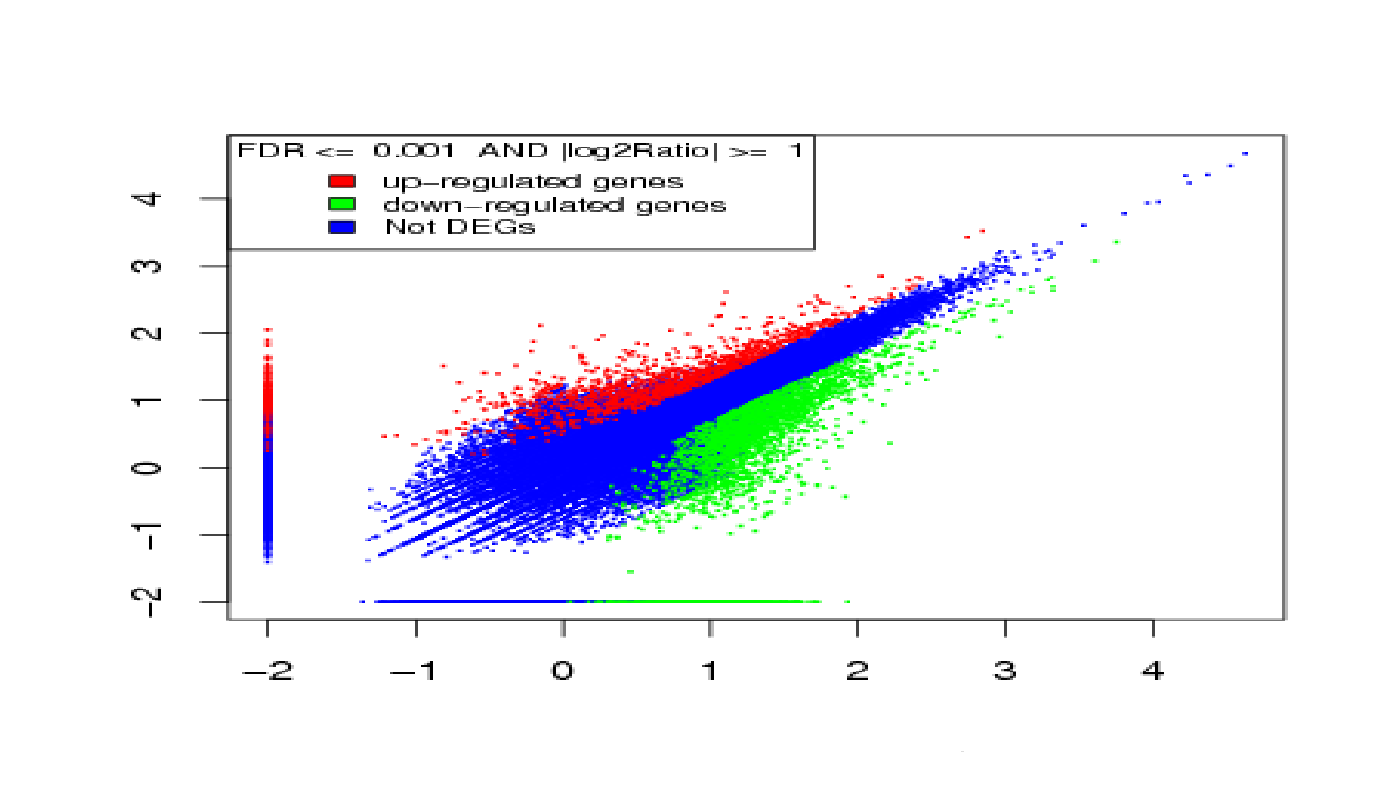


Zheda 622 Log 10 (RPKM) 10(RPKM)

Zheda 622 Log 10 (RPKM) 10(RPKM)

Zheda 622 Log 10 (RPK10(RPKM)

**(C)**

**(B)**

**(A)**

ZS 758 Log 10(RPKM)

Zheda 622 Log 10 (RPKM) 10(RPKM)

ZS 758 Log 10(RPKM)


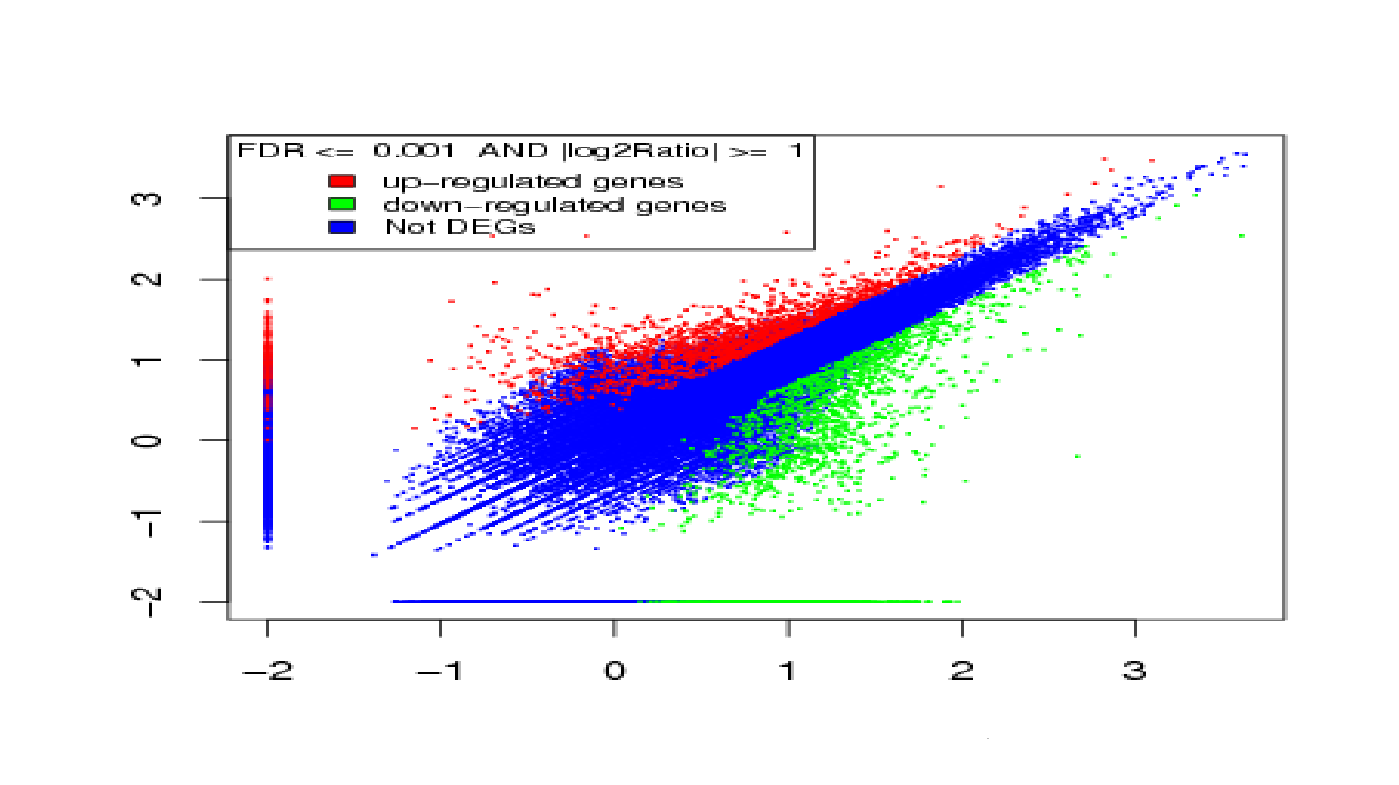


ZS 758 Log 10 (RPKM)

**Fig. S2** Scattered plots shows the pair wise comparison of differentially expressed genes, ZS 758 considered as a control and Zheda 622 as a treatment under the different concentrations. In Figure, (A) represents the control, (B) as Cr 400 µM, and (C) represents the Cr 400 µM + GSH 1 mM.

**(B)**

**(A)**

**Fig. S3** Pie-chart (A) shows the number of up-regulated and down-regulated DEGs in ZS 758/Zheda 622, and (B) Zheda 622/ZS 758, respectively.


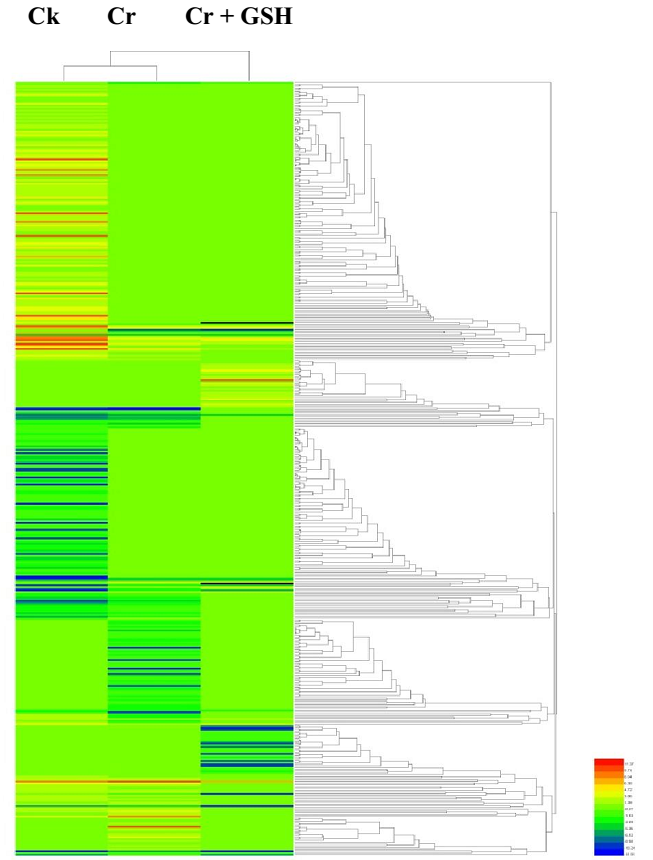

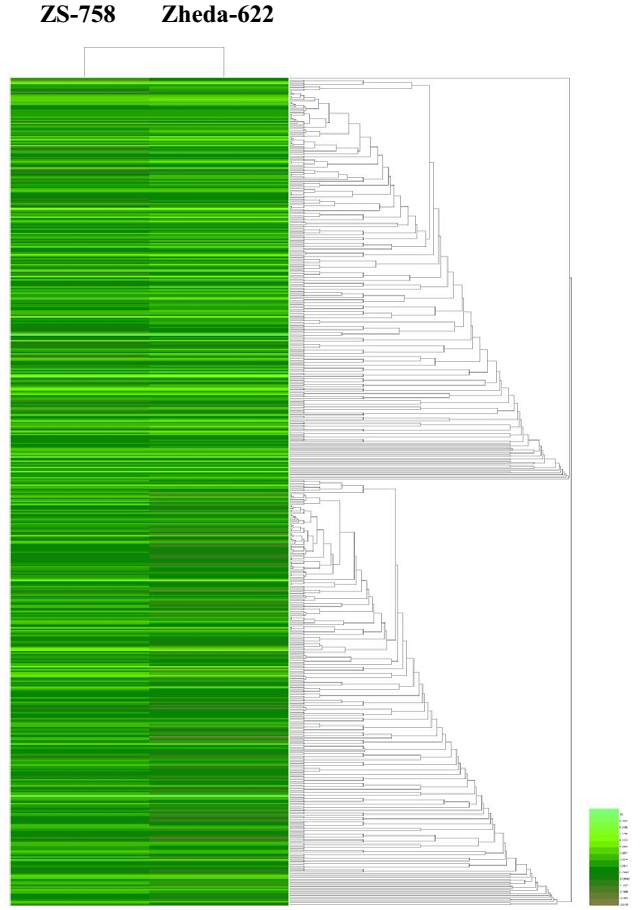


**(B)**

**(A)**

**Fig. S4** HemI hierarchal cluster shows stress responsive relatively differentially expressed genes (DEGs). Diagram (A) shows the DEGs among treatments i.e. Ck, Cr 400 µM, and Cr 400 µM + 1 mM GSH, and (B) between cultivars such as ZS 758 and Zheda 622.


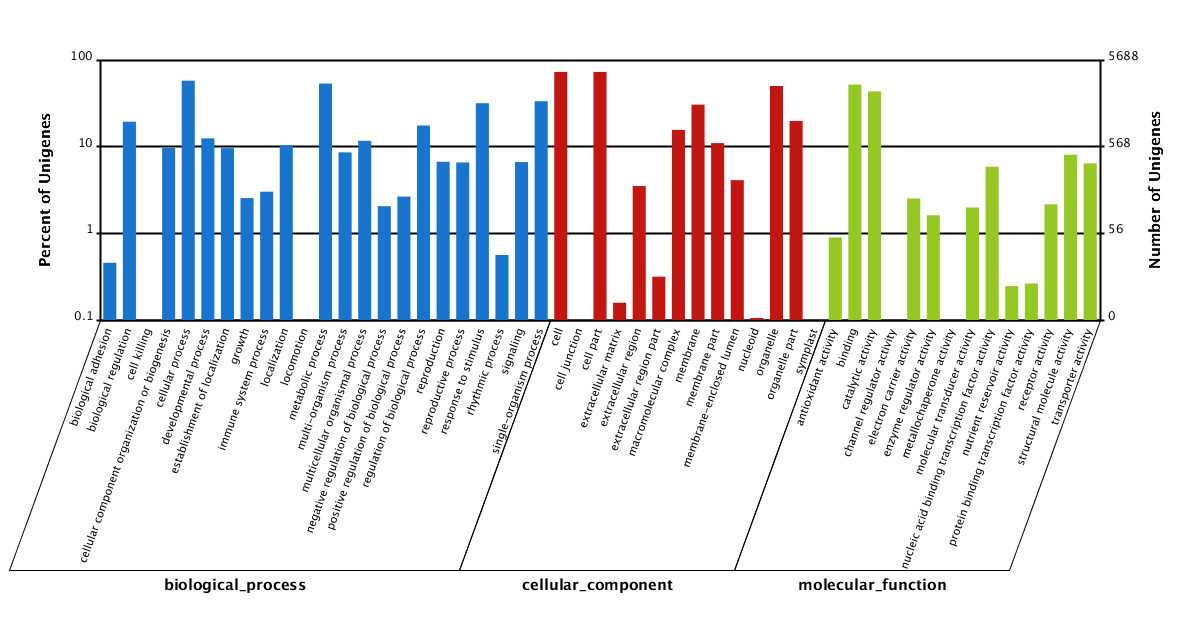


**Fig. S5** Shows the comparative gene ontology functional classification (WEGO) by transcriptome profile analysis in ZS 758 vs Zheda 622. Former cultivar ZS 758 was taken as a control while later cultivar Zheda 622 as a treatment.

**
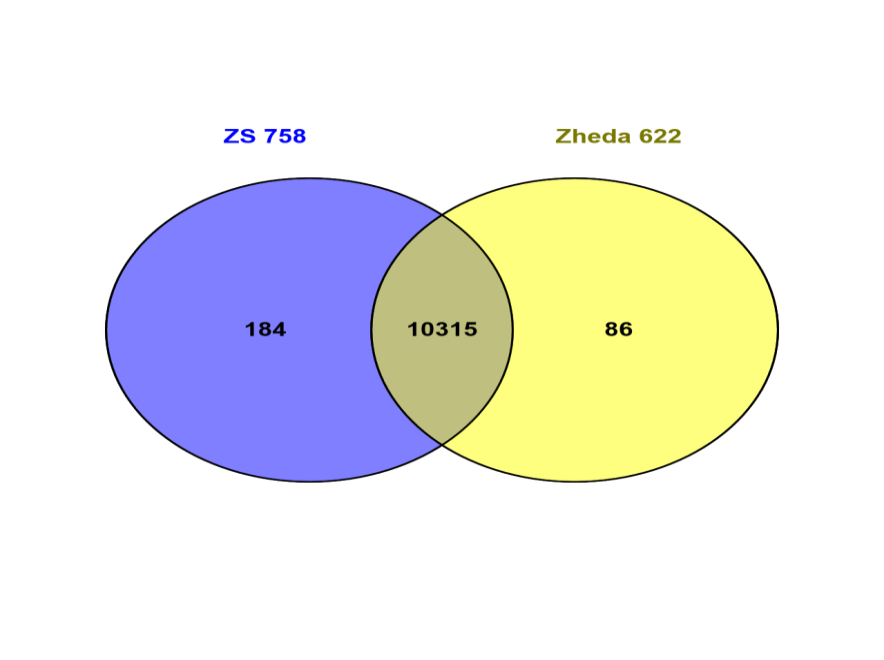

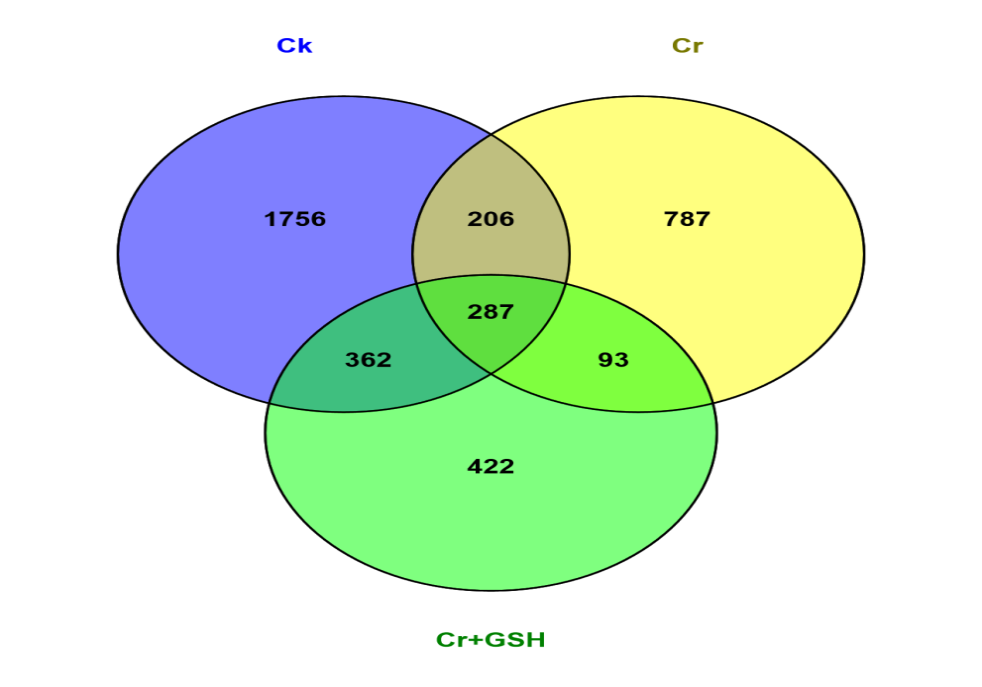
**

**(B)**

**(A)**

**Fig. S6** Diagram showing the transcription factors (TFs) between cultivars and among the treatments. Numbers of each circle show the number of TFs that are uniquely (inside of non-overlapping part) or commonly (inside of overlapping part) regulated. (A) diagram shows the TFs between cultivars, and (B) shows the TFs among the treatments.
